# Mining and application of constitutive promoters from *Rhodosporidium toruloides*

**DOI:** 10.1186/s13568-023-01522-1

**Published:** 2023-02-08

**Authors:** Xiao Guo, Zhenzhen Bai, Yang Zhang, Huimin Zhao, Shuobo Shi

**Affiliations:** 1 Beijing Advanced Innovation Center for Soft Matter Science and Engineering, College of Life Science and Technology, Beijing University of Chemical Engineering, North Third Ring Road 15, Chaoyang District, Beijing, 100029 China; 2 CAS Key Laboratory of Microbial Physiological and Metabolic Engineering, State Key Laboratory of Microbial Resources, Institute of Microbiology, Chinese Academy of Sciences, Beijing, 100101 China; 3 Department of Chemical and Biomolecular Engineering, Carl R. Woese Institute for Genomic Biology, University of IL at Urbana‐Champaign, Urbana, IL 61801 USA; 4 Qinhuangdao Bohai Biological Research Institute, Beijing University of Chemical Engineering, Qinhuangdao, 066000 China

**Keywords:** *R. toruloides*, RNA-seq, Promoters, Biomanufacturing, Synthetic biology

## Abstract

*Rhodosporidium toruloides* is an oleaginous yeast under development with promising industrial applications. Since promoters of different strengths have been demonstrated as an efficient strategy to fine-tune gene expression in synthetic biology, a set of constitutive promoters with strengths varying over 2 orders of magnitude were identified in *R. toruloides* through transcriptome analysis under different growth conditions. Promoter candidates were first cloned and characterized using an enhanced green fluorescent protein (EGFP) as a reporter under eight conditions, and 31 promoters were identified with strength varied from 0.1 to 19.0 folds of the commonly used strong promoter of the glyceraldehyde-3-phosphate dehydrogenase gene (P_GPD1_). The resultant promoters were then used to optimize the linoleic acid biosynthetic pathway in *R. toruloides* in different media, including the use of lignocellulosic hydrolysate as the fermentation substrate, and improved the production of linoleic acid by up to 214.2% in minimal medium, with the highest production of 350.3 mg/L in Yeast Peptone Dextrose medium. This work has enriched the promoter library of *R. toruloides*, and helped develop *R. toruloides* as a platform organism for applications in biomanufacturing and synthetic biology.

**Supplementary Information:**

The online version contains supplementary material available at 10.1186/s13568-023-01522-1.

## Introduction

Microbially produced lipids have emerged as valuable sources of fuels and chemicals that could maintain sustainable development and economic growth (Evans and Ratledge 1983; Gill et al. 1977; Hu et al. 2009). It has been found that the fermented level of lipid production is the key to this shift (Kim et al. 2019; Shi and Zhao 2017) and there has been a growing interest in developing oleaginous microbes for the production of lipids by fermentation. Oleaginous microbes can accumulate lipids of more than 20% of their dry weight (Ratledge and Wynn 2002). Among them, *Rhodosporidium toruloides* can accumulate more than 70% lipids of its dry weight (Jasnos et al. 2005), with a reported lipid production up to 78.7 g/L (Zhao et al. 2011). *R. toruloides* has also been used to produce a high level of fatty alcohols (Fillet et al. 2015), fatty acid methyl ester (FAME) (Thliveros et al. 2014), fatty acid ethyl ester (FAEE) (Zhang et al. 2021), and carotenoids (Buzzini et al. 2007). In addition, its excellent capability for utilizing various cheap feedstocks (Park et al. 2018) makes it a promising production organism for industrial applications.

A promoter could significantly affect the initiation, duration and expression of transcription of the target gene, and is one of the key elements in metabolic engineering and synthetic biology (Ho et al. 2018). During the development of an efficient cell factory, a set of promoters with varying strengths could precisely tune the expression of target genes, and optimize the flux of a target metabolic pathway (Du et al. 2012; Lee et al. 2013; Vogl et al. 2018). This requires a large number of well-characterized promoters with strength varying several orders of magnitude in the target host. However, unlike *Saccharomyces cerevisiae*, only a limited number of well-characterized promoters are available for driving the expression of target genes in the non-model yeast *R. toruloides.* Most reported *R. toruloides* promoters were selected and evaluated based on genes involved in housekeeping or highly expressed pathways. For example, Liu et al. (Liu et al. 2013) reported that P_GPD1_ of *R. toruloides* was able to drive the expression of *EGFP* and the hygromycin phosphotransferase gene (*hpt-3*) with high activity*.* P_GPD1_ is the promoter of the glyceraldehyde-3-phosphate dehydrogenase gene, which has long been considered as a housekeeping gene in many species. Similarly, promoters of six genes involved in lipid accumulation were characterized by a luciferase reporter assay, and one of the identified promoters (P_LDP1_) was adopted to express endogenous gene *DGA1*, which resulted in 21% higher lipid accumulation than that in the strain with P_GPD1_-driven *DGA1* (Liu et al. 2016). In parallel, five constitutive promoters were evaluated by expressing the gene *hpt-3*, and the promoter P_PGI_ showed the highest strength (Wang et al. 2016). Recently, through transcriptional analysis, another twenty constitutive promoters were identified (Nora et al. 2019). Taken together, a total of 31 constitutive promoters have been evaluated in *R. toruloides* so far, with strengths spanning 0.2–11.0 times that of a previously reported strong promoter P_GPD1_ (Liu et al. 2013).

We herein used a combined RNA-seq and biochemical characterization strategy (Liao et al. 2015; Luo et al. 2015) to identify 31 new promoters from *R. toruloides*, which essentially doubled the number of existing promoters in *R. toruloides* and significantly enlarged the promoter strength span. To demonstrate their utility, selected promoters were used to optimize the biosynthetic pathway of linoleic acid, and increased the linoleic acid production to 350.3 mg/L. These promoters should be highly valuable for metabolic engineering of *R. toruloides* for production of value-added products.

## Materials and methods

### Media and culture conditions

*Agrobacterium tumefaciens* AGL1 was grown at 28 °C in either liquid or solid 2YT medium (1.6% tryptone, 1.0% yeast extract, 0.5% NaCl). *E. coli* DH5α was cultured in Luria-Bertani (LB) broth (0.5% yeast extract, 1.0% tryptone, 1.0% NaCl) or on LB agar plates. *R. toruloides* strains were cultured at 30 °C and 250 rpm in following media: Yeast Peptone Dextrose (YPD) medium (1.0% yeast extract, 2.0% peptone, 2.0% glucose), Yeast Peptone Xylose (YPX) medium (1.0% yeast extract, 2.0% peptone, 2.0% xylose), Synthetic Complete Medium (SC) with 20 g/L glucose, and minimal medium (MM) with 20 g/L glucose. The composition of SC and MM medium was reported in a previous study (Zhou et al. 2016). Corncob hydrolysate (CH) medium is composed of corncob hydrolysate and YP solution in a volume ratio of 1:1 and adjust pH to 6.0 with KOH.

To characterize the promoters using *EGFP* as a reporter, strains were pre-cultured overnight at 30 °C in each of the four above-mentioned media. Each pre-culture was then diluted to an optical density at 600 nm (OD_600_) of 0.1, and was cultivated in the corresponding medium at 30 °C for 4–6 h to reach OD_600_ at about 0.6 at the logarithmic growth phase. After incubation for another 36 h, strains entered the stationary growth phase. All strains were harvested and washed once with phosphate buffered saline (PBS) solution for fluorescence analysis under the logarithmic and stationary phase.

For fatty acid production, shake flask fermentation was carried out in different media. Aliquots of *R. toruloides* culture were collected in the stationary phase at 30 °C and 250 rpm.

### Growth curve analysis

For growth curve analysis, *R. toruloides* NP11 that can be accessed from Guangdong Microbial Culture Collection Center (number: GDMCC 2.224) was first inoculated in 5 mL of the five above-mentioned media (YPD, YPX, SC, MM, and CH medium), cultured at 30 °C and 250 rpm for 14 h. Then, 0.1 mL of the above seed culture was transferred to 25 mL of the corresponding medium, and incubated at 30 °C and 250 rpm. During the cultivation, 0.5 mL suspension was sampled for biomass measurement.

OD_600_ values of *R. toruloides* samples were determined using GENESYS 30 Visible Spectrophotometer (Thermo Scientific, USA). The culture with high concentration was diluted to ensure that the OD_600_ is between 0.1 and 0.65.

### Transcriptome analysis by RNA-seq

*R. toruloides* NP11 samples in four media (YPD, YPX, SC and MM medium) and two growth phases (exponential growth and stationary phase) were sent to Novogene Biotech (Beijing, China) for RNA extraction and RNA-seq analysis. All experiments were performed in triplicate.

### Extraction of genomic DNA

The genomic DNA extraction of *R. toruloides* was carried out using the standard phenol/chloroform method (Neumann et al. 1992) with some modifications. Specifically, *R. toruloides* was grown overnight at 30 °C in 2 mL of YPD medium with shaking at 250 rpm. The culture broth was centrifuged at 5000 rpm for 10 min and washed twice with water. Pellets of *R. toruloides* were suspended in 0.2 mL of genomic prep buffer (2.0% Triton X-100, 1.0% SDS, 100 mM NaCl, 10 mM Tris-HCl with pH 8, 1 mM EDTA with pH 8), followed by adding 0.2 mL of 1 mm solid-glass beads (Sigma-Aldrich, Steinheim, Germany) and 0.2 mL of phenol/chloroform/isoamyl alcohol (25:24:1). After vortexing strongly for 30 s, RNase A and Proteinase K with a final concentration of 20 μg/mL and 100 μg/mL were added to remove RNA and protein in the genomic DNA. Finally, the upper solution was treated using the ethanol precipitation method (Green and Sambrook 2016) to obtain the genomic DNA. The concentration was measured by Nanodrop One (Thermo Scientific, USA) and 10 ng of genomic DNA was used as a template for PCR amplification.

### Promoter cloning and plasmid construction

The EGFP reporter gene cassette (P_GPD1_::*EGFP*::T_35S_) was inserted into pKOCAR2 (Koh et al. 2014). The resulting plasmid pKOCAR2-P_GPD1_ was double-digested by FastDigest *Bcu*I and *Nco*I to replace the promoter P_GPD1_ with another candidate promoter sequence.

All candidate promoter sequences were amplified by PCR using *R. toruloides* NP11 genomic DNA as the template unless indicated otherwise. Sequence 1000 bp upstream of the start codon was chosen as potential promoter regions. In addition, two previously reported promoters of *R. toruloides* were used as controls, including P_FAS1_ (Liu et al. 2016) and P_TPI_ (Wang et al. 2016).

A series of plasmids including pKOCAR2-Sn (n = 1 to 60), pKOCAR2-P_FAS1_, and pKOCAR2-P_TPI_ were constructed for evaluation of promoter strength (Additional file 1: Table S1). Briefly, the *Bcu*I/*Nco*I digested pKOCAR2-P_GPD1_ vector and each amplified promoter fragment were mixed with a molar ratio of 1:3 in a PCR tube, and 5 μL 2 × NEBuilder^®^ HiFi DNA Assembly Master Mix was added to make a total volume of 10 μL. The mixture was incubated at 50 °C for 2 h for DNA assembly.

Plasmids for optimizing the production of linoleic acid were constructed as follows. First, promoter P_RT12_, *FAD9* gene, promoter P_RT14_, and *FAD12* gene were amplified from the *R. toruloides* NP11 genomic DNA, and the terminator T_SV40_ and T_35S_ sequences were amplified using pKOCAR2-P_GPD1_ as the template. Then, gene cassettes of P_RT12_ promoter-controlled *FAD9*-expressing cassette (P_RT12_-*FAD9*-T_SV40_) and P_RT14_ promoter-controlled *FAD12* cassette (P_RT14_-*FAD12*) were constructed by fusion PCR. Plasmid pKOCAR2-P_GPD1_ was double-digested by FastDigest *Bcu*I and *Eco*RV, removing the P_GPD1_-*EGFP* fragment and then assembled with the above two gene expression cassettes by the NEBuilder method to form plasmid pKOCAR2-S56. Plasmids from pKOCAR2-S57 to pKOCAR2-S60 that contain different expression cassettes were also constructed using the same method. The oligonucleotides used are listed in Additional file 1: Table S2. All strains and plasmids are listed in the Additional file 1: Table S1.

### Fluorescence measurement

Strains containing different EGFP expression cassettes were analyzed with a fluorescence microscope (Olympus IX73; Olympus, Tokyo, Japan). First, an image with a magnification of 40 × in bright filed (BF) was photographed. Subsequently, EGFP fluorescence was observed and photographed using the channel of fluorescein isothiocyanate (FITC) with the excitation (ex) at 490 nm and the emission (em) at 518 nm. An automatic exposure time was set. In parallel, a 200 μL culture of *R. toruloides* containing different EGFP expression cassettes were transferred to Corning^™^ Clear Polystyrene 96-Well Microplates and Costar^®^ 96-Well Black Polystyrene Plate (Corning Incorporated, USA) for the measurement of biomass and fluorescence. The biomass and fluorescence were measured using EnSpire^®^ Multimode Plate Reader (PerkinElmer, USA) under 600 nm or 485(ex)/525(em) nm, respectively. The fluorescence signal was normalized to the cell density to make the data comparable.

### Metabolite analysis

HPLC (Shimadzu, Japan) equipped with an Aminex HPX-87H column (Bio-Rad, USA) was used to detect the concentrations of glucose, xylose, and arabinose in CH medium. After centrifugation at 12000 rpm, the supernatant of *R. toruloides* culture was determined by RI-101 Refractive Index Detector, using 0.5 mM H_2_SO_4_ as the mobile phase at a flow rate of 0.4 mL/min.

GC-MS QP2020 (SHIMADZU, Kyoto, Japan) equipped with Rtx-5MS column (30 m × 0.25 mm, 0.25 μm film thickness) (Restek, Bellefonte, PA, USA) was applied to detect the lipid in *R. toruloides* culture. Lipid extraction and transmethylation were performed in one step according to a previous method (Khoomrung et al. 2012) and the GC-MS program for fatty acid methyl esters (FAMEs) analysis was described elsewhere (Zhang et al. 2021).

## Results

### Transcriptome analysis of *R. toruloides* under different conditions

*R. toruloides* NP11 was cultured using Yeast Peptone Dextrose (YPD), Yeast Peptone Xylose (YPX), Synthetic Complete Medium (SC), and minimal medium (MM) media. Through analyzing the transcriptome data of *R. toruloides* in these four media and two individual growth phases (logarithmic and stationary growth phase) (Additional file 1: Fig. S1), approximately 8233 transcripts were identified for each condition. The promoters of the top-expressed 15 genes in each condition were selected as the promoter candidates with high strength. Selected genes are shown in Additional file 1: Table S3. After removing duplicates from the list, a total of 52 promoters were selected, which were marked in bold (Additional file 1: Table S3). Next, due to the failure of cloning, a total of 49 promoter sequences were amplified and cloned for strength evaluation using enhanced green fluorescent protein (EGFP) as the reporter (Additional file 1: Fig. S2a). Meanwhile, three reported promoters from *R. toruloides*, including P_GPD1_ (Koh et al. 2014), P_FAS1_ (Liu et al. 2016) and P_TPI_ (Wang et al. 2016), were also cloned to drive the expression of EGFP as positive controls.

### Characterization of EGFP fluorescence intensity and promoter strength

EGFP expression cassettes driven by candidate and control promoters were integrated into the *CAR2* locus, resulting in white colonies due to the deletion of *CAR2* gene (Additional file 1: Fig. S2b) (Koh et al. 2014). Recombinant strains were firstly verified through the fluorescence microscope analysis (Additional file 1: Fig. S2c). As shown in Additional file 1: Table S4, 11 strains did not show EGFP fluorescence and 7 strains exhibited very weak fluorescence; 34 strains (including 3 controls with *EGFP* driven by P_FAS1_, P_TPI_, and P_GPD1_ and 31 strains with *EGFP* driven by new identified 31 promoters) showed strong fluorescence. Sequence analysis of the new identified 31 promoters revealed several identifiable core promoter elements. For example, one putative CAAT motif that worked as regulators binding site was found in the sequence of some promoters such as P_RT2_, P_RT33_, P_RT36_, and P_RT38_, and a possible GC box (GGGCGGG) that played a role in the recognition of the promoter region was found in several promoters such as P_RT13_ and P_RT29_. Moreover, a pyrimidine-rich region (CT box, − 10 to − 90 bp from the translational start codon) was found in all identified 31 promoters, consistent with previous reports (Liu et al. 2015). On the other hand, conspicuous introns, acrossing the untranslated regions and coding sequences boundary, may exist in the failed promoters for driving EGFP (Additional file 1: Table S5).

**Fig. 1:**
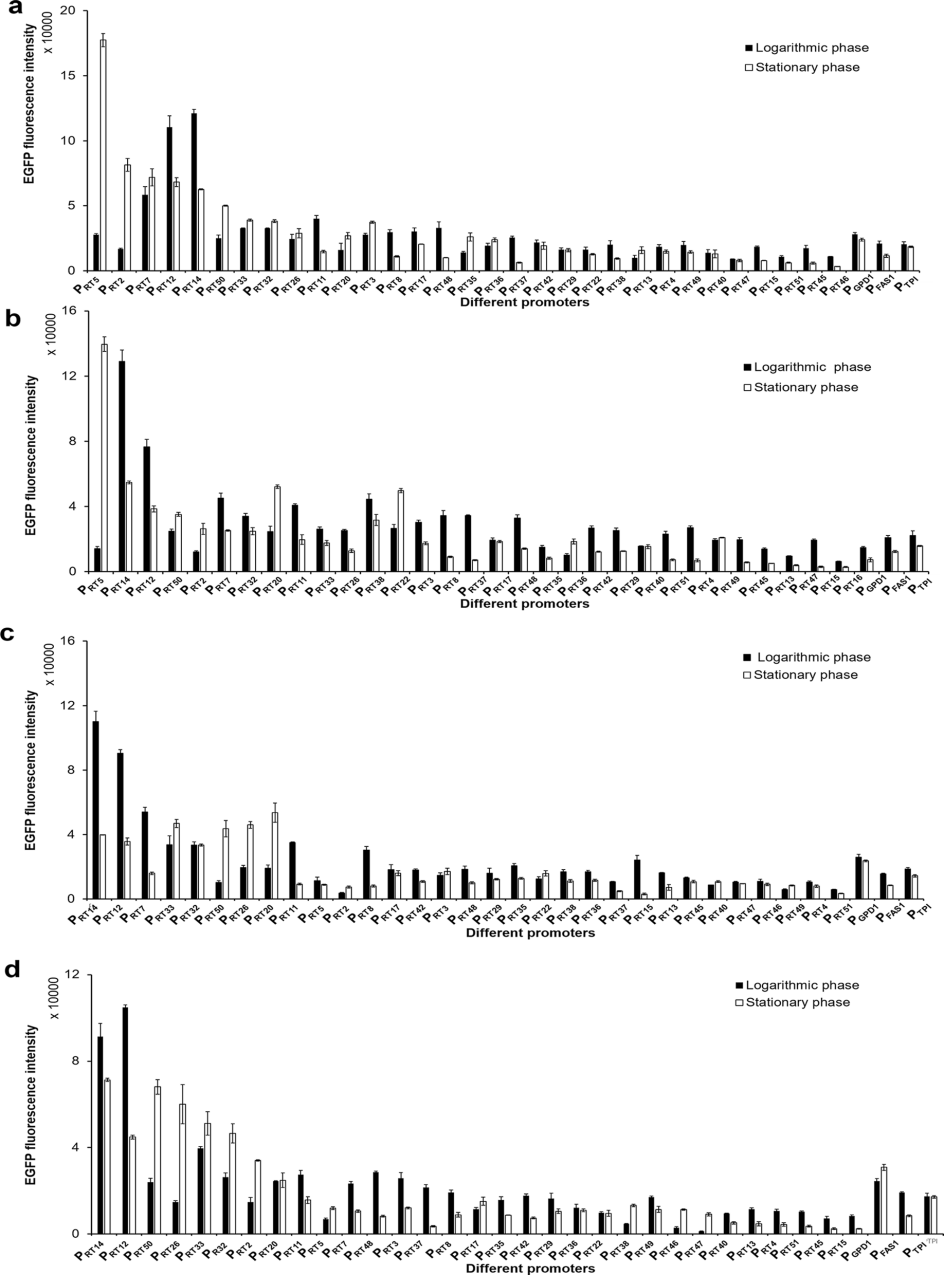
EGFP fluorescence intensities expressed by different promoters in four types of media under different growth phases. **a** YPD medium; **b** YPX medium; **c** SC medium; **d** MM medium. Solid columns represent the logarithmic growth phase and hollow columns represent the stationary phase. The corresponding sampling time for logarithmic and stationary growth phase is: 23 h and 48 h for YPD, 26 h and 60 h for YPX, 26 h and 48 h for SC, 20 h and 48 h for MM medium, respectively. Data were derived from triplicates

EGFP fluorescence intensities of the 34 recombinant strains were then determined under different conditions (Fig. 1 and Table 1). We compared the strength of these promoters with P_GPD1_ under various culture conditions and came to the following conclusion: the strength of 31 promoters is 0.1–19.0 times that of P_GPD1_. It is worth noting that promoter P_GPD1_ commonly viewed as a strong promoter was classified as medium-strong in this promoter set. Through heatmap analysis (Additional file 1: Fig. S3), we suggested that promoters such as P_RT12_ and P_RT14_ were extremely strong promoters in all four types of culture medium and the strength of some promoters such as P_RT5_ and P_RT2_ varied significantly in different media. We also observed that the phases of growth gave a different effect on the strength. For example, P_RT12_ and P_RT14_ performed a slightly stronger strength in the logarithmic stage than that in the stationary stage. In contrast, the strengths of P_RT5_ and P_RT2_ in the stationary phase were much higher than that in the logarithmic phase, which can be up to 9 times higher in the YPD medium. These results suggest that the selection of appropriate promoters could confer appropriately medium- or growth- responsive expression.

**Table 1:** Description of identified 31 promoters from *R. toruloides*.

Promoter name	ID of the corresponding gene	Length of the corresponding gene (bp)	Gene function
P_RT2_	27368376	1029	L-malate dehydrogenase
P_RT7_	27371855	1932	Heat shock 70 kDa protein 1/8
P_RT5_	27365962	339	Hypothetical protein
P_RT32_	27367759	1038	Glyceraldehyde-3-phosphate dehydrogenase
P_RT14_	27369110	1386	Elongation factor EF-1 alpha subunit
P_RT50_	27366188	333	60S acidic ribosomal protein p2
P_RT12_	27369809	1983	Molecular chaperones mortalin/PBP74/GRP75, HSP70 superfamily
P_RT33_	27364336	1341	Enolase
P_RT20_	27371047	1587	Aromatic amino acid aminotransferase I
P_RT11_	27364645	444	Large subunit ribosomal protein L27Ae
P_RT26_	27369450	5295	ABC bile acid transporter
P_RT3_	27369119	927	Mitochondrial ADP/ATP carrier
P_RT8_	27371881	1122	60S ribosomal protein l2
P_RT37_	27368163	2457	Hypothetical protein
P_RT35_	27365895	1752	Amino acid transporter
P_RT42_	27370537	642	Hypothetical protein
P_RT48_	27366763	327	NP11 60S acidic ribosomal protein p1
P_RT17_	27371704	2304	5-methyltetrahydropteroyltriglutamate-homocysteine S-methyltransferase
P_RT36_	27369851	1512	Aldehyde dehydrogenase (NAD)
P_RT29_	27364309	2817	Aminopeptidase 2
P_RT13_	27370419	1374	Citrate synthase
P_RT15_	27364137	1068	Hypothetical protein
P_RT38_	27370093	1647	MFS monosaccharide transporter
P_RT22_	27371731	3132	Glutamate dehydrogenase (NAD^+^)
P_RT4_	27367219	1620	F0F1-type ATP synthase, beta subunit
P_RT40_	27367075	1080	Alcohol dehydrogenase
P_RT45_	27365879	3003	Plasma membrane H^+^- transporting ATPase
P_RT46_	27366699	1662	Hypothetical protein
P_RT47_	27371760	1191	Alternative oxidase
P_RT49_	27368829	567	60S ribosomal protein l19
P_RT51_	27366128	465	Small subunit ribosomal protein S27Ae

Corresponding gene ID was obtained according to our transcriptome sequencing data, and it can be accessed via the NCBI database (https://www.ncbi.nlm.nih.gov/gene/)

The strengths of some previously known promoters and five representative promoters we identified are compared in Additional file 1: Table S6. In our work, a more representative library was characterized with a greater dynamic range of the promoter strength, which provides more possibilities to regulate gene expression in *R. toruloides*. In our identified promoters, through “gene function annotation”, several promoters were found to possess the same function as the previous reported promoters (Additional file 1: Table S7). However, due to the different definition in the promoter regions, there are differences in the DNA sequence, indicating they are not the same promoters.

### Application of the identified promoters for combinatorial pathway optimization

**Fig. 2:**
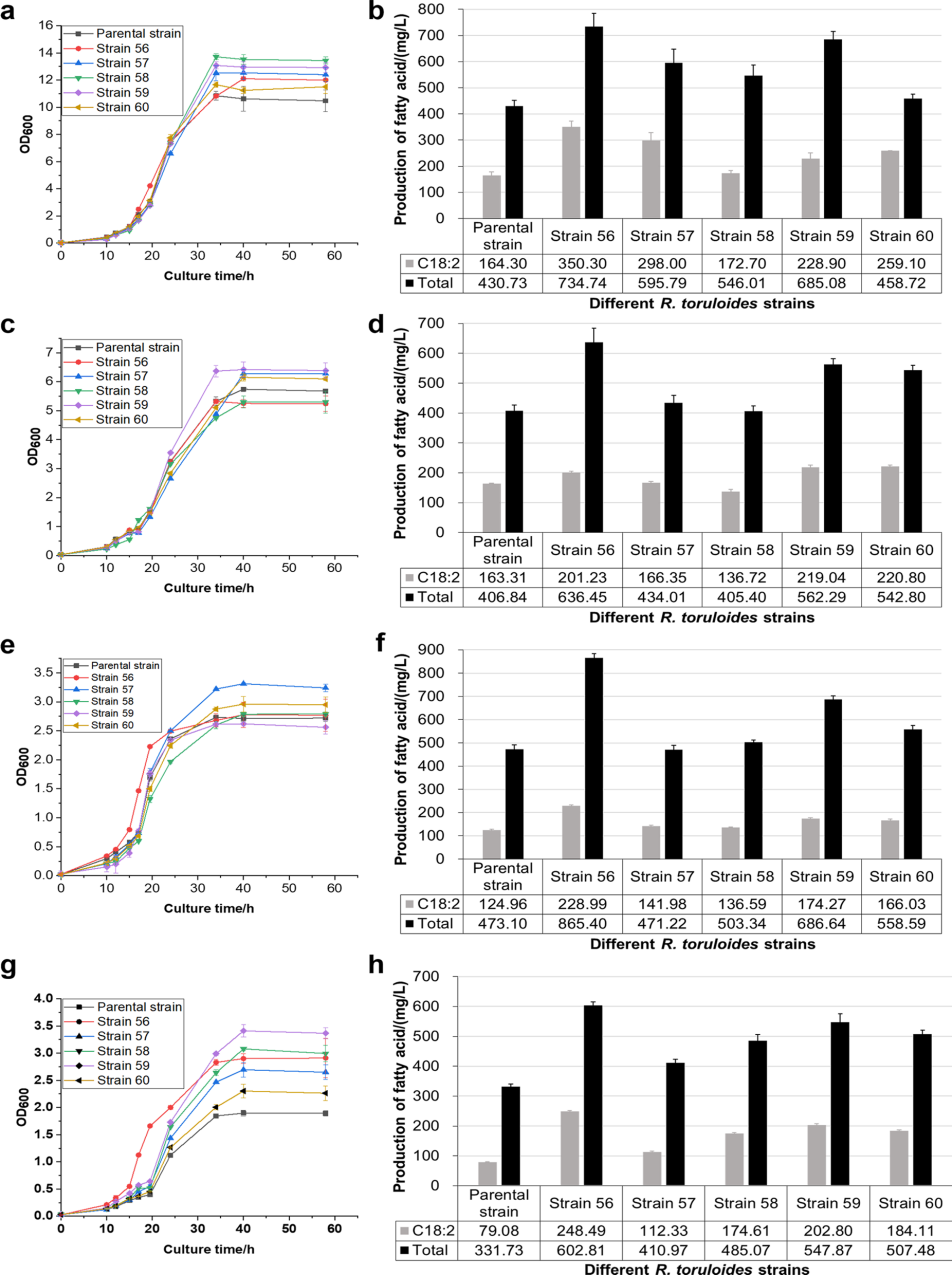
Growth status and fatty acid production of different *R. toruloides* strains in 4 kinds of media. Cell growth analysis of *R. toruloides* cultured in **a** YPD, **c** YPX, **e** SC, and **g** MM medium. Parental strain, *R. toruloides* NCYC 1585; strain 56, *R. toruloides* NCYC 1585 with plasmid pKOCAR2-S56; strain 57, *R. toruloides* NCYC 1585 with plasmid pKOCAR2-S57; strain 58, *R. toruloides* NCYC 1585 with plasmid pKOCAR2-S58; strain 59, *R. toruloides* NCYC 1585 with plasmid pKOCAR2-S59; strain 60, *R. toruloides* NCYC 1585 with plasmid pKOCAR2-S60. Production of fatty acid by *R. toruloides* strains in **b** YPD, **d** YPX, **f** SC, and **h** MM medium. C18:2, linoleic acid. Total, total fatty acid. Data were derived from triplicates

Linoleic acid is one of the most highly consumed polyunsaturated fatty acid in human diet. A series of strains have been reported for the production of linoleic acid, with greatly varied titers (Lamers et al. 2019; Wu et al. 2021). Thus, we applied the identified promoters with different strengths to optimize the biosynthetic pathway of linoleic acid in *R. toruloides*, which consists of delta (9) fatty acid desaturase gene (*FAD9*) and delta (12) fatty acid desaturase gene (*FAD12*) (Additional file 1: Fig. S4a). Specifically, two extremely high-strength promoters (P_RT12_ and P_RT14_) and a high-strength promoter (P_RT46_) were selected from these 31 identified promoters to combinatorically express *FAD9* and *FAD12*, while promoter P_GPD1_ was used as a control (Additional file 1: Fig. S4b). The growth curves and fatty acid production of these engineered strains (strain 56-strain 60) and their parental strain *R. toruloides* NCYC 1585 were shown in Fig. 2. Compared to the parental strain, most engineered strains with the overexpression of *FAD9* and *FAD12* grew slightly faster and produced more linoleic acid (Fig. 2).

Overall, the best-performing strain is strain 56 with the use of two extremely high-strength promoters (P_RT12_ and P_RT14_), with the linoleic acid production increased from 164.3 mg/L of the parent strain to 350.3 mg/L in YPD medium, and from 79.08 mg/L of the parent strain to 248.5 mg/L in MM medium (a 214.2% improvement). Furthermore, strain 56 also gave the highest linoleic acid production in SC medium, while strains 56, 59 and 60 showed a similar ability for linoleic acid production in YPX medium. This result also proved that in these widely used media, the strain with the best combination of our identified promoters gave higher production of linoleic acid than strain 60 with the use of two P_GPD1_ promoters, and achieved the optimized expression of a biosynthetic pathway. Overall, the data illustrated the feasibility of using promoters with varying strengths in optimizing metabolic pathways in *R. toruloides*.

**Fig. 3:**
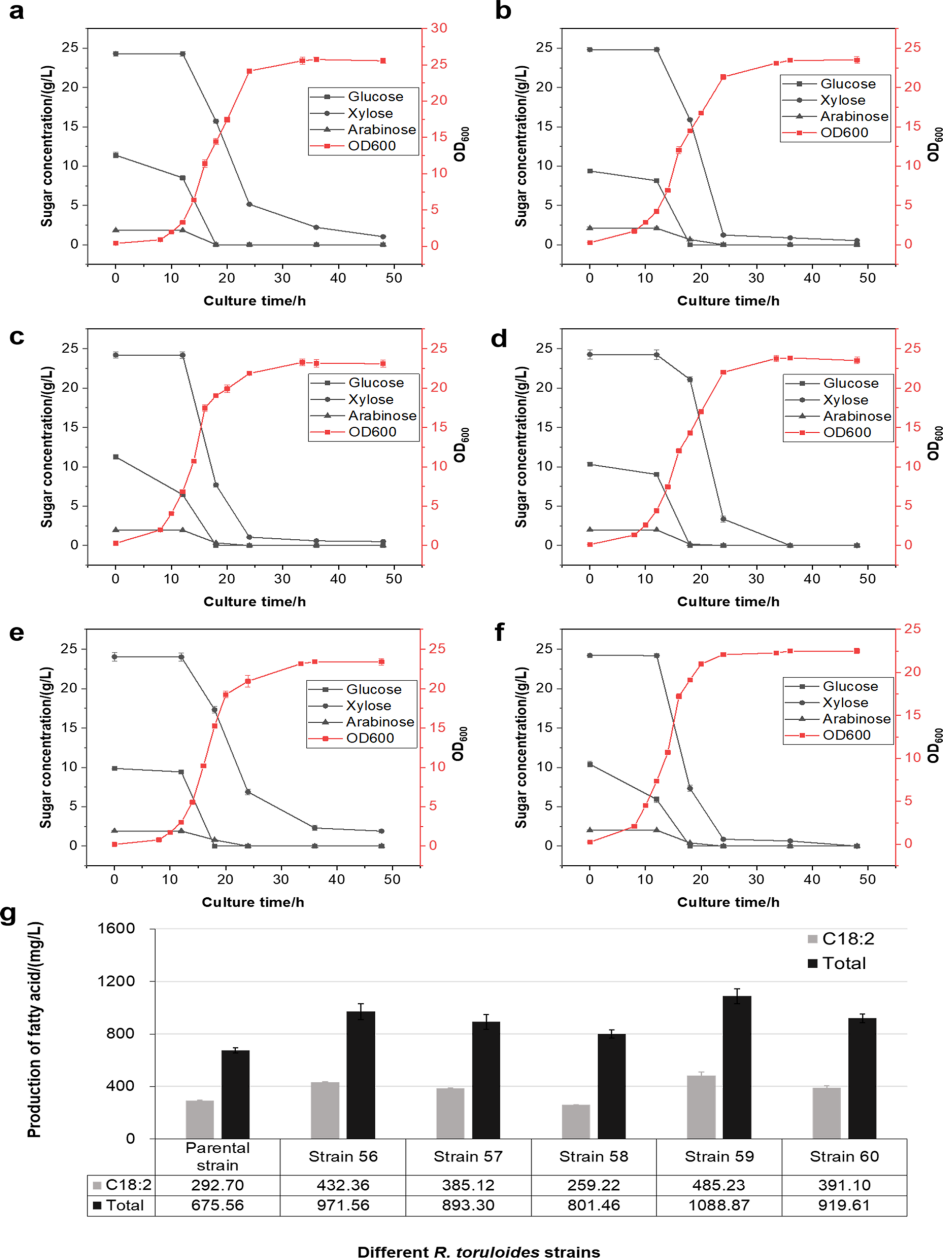
Cell growth and fatty acid production of different *R. toruloides* strains in CH medium. Sugar consumption and growth curves of: **a** parental strain, **b** strain 56, **c** strain 57, **d** strain 58, **e** strain 59, **f** strain 60 in CH medium. Parental strain, *R. toruloides* NCYC 1585; strain 56, *R. toruloides* NCYC 1585 with plasmid pKOCAR2-S56; strain 57, *R. toruloides* NCYC 1585 with plasmid pKOCAR2-S57; strain 58, *R. toruloides* NCYC 1585 with plasmid pKOCAR2-S58; strain 59, *R. toruloides* NCYC 1585 with plasmid pKOCAR2-S59; strain 60, *R. toruloides* NCYC 1585 with plasmid pKOCAR2-S60. Black squares, black dots, black triangles and red squares represent glucose, xylose, arabinose and OD_600_, respectively. **g** Production of fatty acid by *R. toruloides* strains in CH medium. C18:2, linoleic acid. Total, total fatty acid. Data were derived from triplicates

Since *R. toruloides* has the ability to utilize low-cost lignocellulosic biomass such as corncob hydrolysates (Park et al. 2018), the above constructed series of engineered strains were evaluated for linoleic acid production with the use of corncob hydrolysate (named CH medium) as the fermentation substrate. The results showed that all engineered strains of *R. toruloides* were able to maintain a good growth from corncob hydrolysate (Fig. 3a). Released sugars from corncob hydrolysate included xylose, glucose and arabinose, and more than 80% of them could be quickly utilized within 30 h. Noticeably, the initial concentration of xylose was much higher than that of glucose released from the corncob hydrolysate, but glucose was found as the first consumed sugar. Among these engineered strains, strain 59 produced more linoleic acid with the use of two P_RT46_ promoters (Fig. 3b). Thus, it is speculated that P_RT46_ promoter may contribute better gene expression in medium using mixed sugars (glucose and xylose).

## Discussion

*R. toruloides* is an important non-model oil-producing microorganism. Under the limitation of nitrogen, the accumulation of oil production can reach 70% of its dry cell weight (Li et al. 2007). Various fatty acid derived fuels and chemicals, such as FAEE (Zhang et al. 2021) and polyunsaturated fatty acids (Tsai et al. 2019), have been produced in *R. toruloides* by the introduction of different metabolic pathways. With the availability of well-characterized promoters in model organisms, it has been demonstrated to successfully construct the efficiently expressed and well-balanced metabolic network by regulating the transcription level. Thus, this work provided more promoters with different strengths under various conditions by RNA-seq analysis in *R. toruloides*, and further used them for optimizing the production of linoleic acid.

As described in Additional file 1: Table S6, the strengths of some known promoters were compared with promoters reported in this work. Initially, the strength of promoter P_GPD1_ was evaluated in YPD medium by *EGFP* reporter assay and classified as a strong promoter of *R. toruloides* (Liu et al. 2013). Later, it was found that P_TPI_ was about 1.2 times stronger than P_GPD1_, assessed by driving *hpt-3* gene expression in YPD medium (Wang et al. 2016). In parallel, P_FAS1_ was characterized as possessing 20–90% of the strength of P_GPD1_ using luciferase reporter gene *RtLUC2* in YPD medium (Liu et al. 2016). In our study, the fluorescence intensity driven by the promoter P_FAS1_ was 73%-77% compared with that of P_GPD1_, which was consistent with the above-mentioned previously reported results (Liu et al. 2016). Moreover, the strength of novel promoters we identified showed a varied strength ranging from 0.1 to 19.0 times the P_GPD1_ strength. The weakest promoter is P_RT45_ in MM medium (0.1-fold of P_GPD1_ strength), and the strongest is P_RT5_ in YPX medium (19.0-fold of P_GPD1_ strength). Furthermore, P_RT12_ and P_RT14_ gave very strong promoter strength in the four media tested above. In Table 1, it showed gene functions of these 31 promoters. Genes associated with the identified promoters have a broad range of functions, including metabolic enzymes (e.g. P_RT2_), transporters (e.g. P_RT35_), protein translation (e.g. P_RT14_), and global regulators (e.g. P_RT12_). We noticed that the gene functions corresponding to P_RT12_ and P_RT14_ are housekeeping genes, which could be the reason for their high transcriptional strength. For example, the corresponding gene of P_RT14_ is supposed to encode elongation factor EF-1 alpha, which is responsible for the efficient transport of amino-acyl transfer RNAs (tRNAs) to ribosome, and the promoter of this gene is also used as a common strong promoter in *S. cerevisiae* and *Yarrowia lipolytica* (Müller et al. 1998). However, there is a lack of relevant studies in many promoters identified in this study. How the original gene functions affected the performance of these promoters should be carried out in the future and compared with promoters of homologous genes that identified in other species, which may contribute a novel guideline for mining promoters.

A similar work has been reported with *R. toruloides*, identifying 20 constitutive promoters, 12 monodirectional promoters and 8 bidirectional promoters (Nora et al. 2019). Compared with this study, seven promoters we identified overlapped with their reported promoters based on protein ID. However, the sequences of these promoters are not the same, due to the different definition in the promoter regions. Detailed descriptions of these promoters are listed in Additional file 1: Table S7. In our work, a more representative library was characterized with a greater dynamic range of the promoter strength, which provides more options and possibilities for *R. toruloides* to regulate gene expression. The approach we used could also allow for the rapid discovery of endogenous promoters in other non-model organisms.

Linoleic acid is the most highly consumed polyunsaturated fatty acid in human diet. A series of strains have been reported for the production of linoleic acid, with greatly varying titers (Lamers et al. 2019; Wu et al. 2021). It has been shown that fine-tuning gene expression is an efficient strategy for pathway optimization. Here, as a case study, the biosynthetic pathway of linoleic acid was optimized using these identified promoters with different strength in *R. toruloides*. Several engineered strains with improved linoleic acid production were obtained through a combinatorial expression of the pathway genes *FAD9* and *FAD12* in different media, including the use of corncob hydrolysate in the medium, noticing the practical significances to use lignocellulose hydrolysates owing to their low costs, renewability, and availability (Huang et al. 2012; Yu et al. 2014). After optimization, there is 65.8% increase in the linoleic acid production to 485.23 mg/L, demonstrating the value of fine-tuning gene expression via a promoter library. With the broader dynamic range of our promoter library, it is expected that it would serve as a generally applicable tool for genetic engineering of *R. toruloides*.

In conclusion, the discovery and application of novel promoters has been explored in many hosts for synthetic biology applications. In this study, we established a library of promoters with a large dynamic range through RNA-seq analysis. After the promoter strength characterization using EGFP as the reporter, 31 promoters were identified with strength varying from 0.1 to 19.0 folds of the commonly used strong promoter P_GPD1_, which not only doubled the number of existing promoters of *R. toruloides* but also significantly enlarged the strength span. Subsequently, the identified promoters were applied to optimize the biosynthetic pathway of linoleic acid in different media, and improved the production to varied extent in different media compared with overexpression using the commonly used strong promoter P_GPD1_. This library of promoters could be useful for metabolic engineering and synthetic biology applications in *R. toruloides*. The approach we used could also allow for the rapid discovery of endogenous promoters in other non-model organisms.

## Supplementary Information

**Additional file 1: Fig S1.** Cell growth analysis of *R. toruloides* NP11 cultured in different media. **Fig S2.** Plasmid construction and fluorescence images of engineered *R. toruloides*. **Fig S3.** Expression level of *EGFP* controlled by identified 31 promoters. **Fig S4.** Combinatorial pathway engineering of linoleic acid production in *R. toruloides*. **Table S1.** Strains and plasmids used in this study. **Table S2.** All primers used in this study. **Table S3.** Gene ID (description of the ID can be found in NCBI database: https://www.ncbi.nlm.nih.gov/gene/) of the top 15 genes with the highest expression levels under different culture conditions at logarithmic growth phase and stationary growth phase: 23 h and 48 h for YPD (YPD23 and YPD48), 26 h and 60 h for YPX (YPX26 and YPX60), 26 h and 48 h for SC (SC26 and SC48), 20 h and 48 h for MM (MM20 and MM48). A total of 52 candidate genes were obtained after removing the duplicates, marked in bold. By blasting these genes in NCBI (https://www.ncbi.nlm.nih.gov/assembly/GCA_000320785.2), we obtained the scaffold sequences where these genes are located. **Table S4.** Summary of the cloning and evaluation results of 52 candidate promoters. Among them, 3 promoter sequences were not successfully amplified from *R. toruloides* genome; 11 promoters did not generate any EGFP fluorescence; 7 promoters drove very weak EGFP fluorescence expression and no further study was performed; 31 promoters driving high EGFP expression were further investigated. The ID of the promoter’s corresponding gene can be obtained on NCBI website. **Table S5.** The sequence analysis of promoters failed to drive EGFP expression. The highlighted font are the possible G/GT and AG/G splice sites of introns (Chung et al., 2006) between the 3' end of the promoter (the black font) and the first 22 bp of *EGFP* gene (the green font). **Table S6.** Summary of the relative strengths of known promoters and our identified five new promoters native to *R. toruloides*. The ID of the promoter’s corresponding gene can be accessed via the NCBI database (https://www.ncbi.nlm.nih.gov/gene/). **Table S7.** List of the overlapped promoters identified in this study with the previous reported work.

## Data Availability

The authors confirm that the datasets supporting the findings and the conclusions of this study are available within the article and its supplementary information file. The data reported in this paper have been deposited in the OMIX, China National Center for Bioinformation/Beijing Institute of Genomics, Chinese Academy of Sciences (https://ngdc.cncb.ac.cn/omix accession no. OMIX001217).
