# Supplementary material for: Mining and application of constitutive promoters from Rhodosporidium toruloides
Source: AMB Express. 2023 Feb 8;13:17. doi: 10.1186/s13568-023-01522-1 (PMC9908808; doi:10.1186/s13568-023-01522-1)
Supplement: Supplementary file 1 — Additional file 1: Fig S1. Cell growth analysis of R. toruloides NP11 cultured in different media. Fig S2. Plasmid construction and fluorescence images of engineered R. toruloides. Fig S3. Expression level of EGFP controlled by identified 31 promoters. Fig S4. Combinatorial pathway engineering of linoleic acid production in R. toruloides. Table S1. Strains and plasmids used in this study. Table S2. All primers used in this study. Table S3. Gene ID (description of the ID can be found in NCBI database: https://www.ncbi.nlm.nih.gov/gene/) of the top 15 genes with the highest expression levels under different culture conditions at logarithmic growth phase and stationary growth phase: 23 h and 48 h for YPD (YPD23 and YPD48), 26 h and 60 h for YPX (YPX26 and YPX60), 26 h and 48 h for SC (SC26 and SC48), 20 h and 48 h for MM (MM20 and MM48). A total of 52 candidate genes were obtained after removing the duplicates, marked in bold. By blasting these genes in NCBI (https://www.ncbi.nlm.nih.gov/assembly/GCA_000320785.2), we obtained the scaffold sequences where these genes are located. Table S4. Summary of the cloning and evaluation results of 52 candidate promoters. Among them, 3 promoter sequences were not successfully amplified from R. toruloides genome; 11 promoters did not generate any EGFP fluorescence; 7 promoters drove very weak EGFP fluorescence expression and no further study was performed; 31 promoters driving high EGFP expression were further investigated. The ID of the promoter’s corresponding gene can be obtained on NCBI website. Table S5. The sequence analysis of promoters failed to drive EGFP expression. The highlighted font are the possible G/GT and AG/G splice sites of introns (Chung et al., 2006) between the 3' end of the promoter (the black font) and the first 22 bp of EGFP gene (the green font). Table S6. Summary of the relative strengths of known promoters and our identified five new promoters native to R. toruloides. The ID of the promoter’s correspondi [file 13568_2023_1522_MOESM1_ESM.docx]

**Supplementary Information**

**Mining and Application of Constitutive Promoters from *Rhodosporidium toruloides***

Xiao Guo^1^, Zhenzhen Bai^1^, Yang Zhang^1, 2^, Huimin Zhao^3*^, Shuobo Shi^1^^,4*^

^1^Beijing Advanced Innovation Center for Soft Matter Science and Engineering, College of Life Science and Technology, Beijing University of Chemical Engineering, Beijing 100029, China

^2^CAS Key Laboratory of Microbial Physiological and Metabolic Engineering, State Key Laboratory of Microbial Resources, Institute of Microbiology, Chinese Academy of Sciences, Beijing 100101, China

^3^Department of Chemical and Biomolecular Engineering, Carl R. Woese Institute for Genomic Biology, University of Illinois at Urbana‐Champaign, Urbana, IL 61801, USA

^4^ Qinhuangdao Bohai Biological Research Institute, Beijing University of Chemical Engineering, Qinhuangdao 066000, China

**Correspondence:**

*E-mail: zhao5@illinois.edu

*E-mail: shishuobo@mail.buct.edu.cn


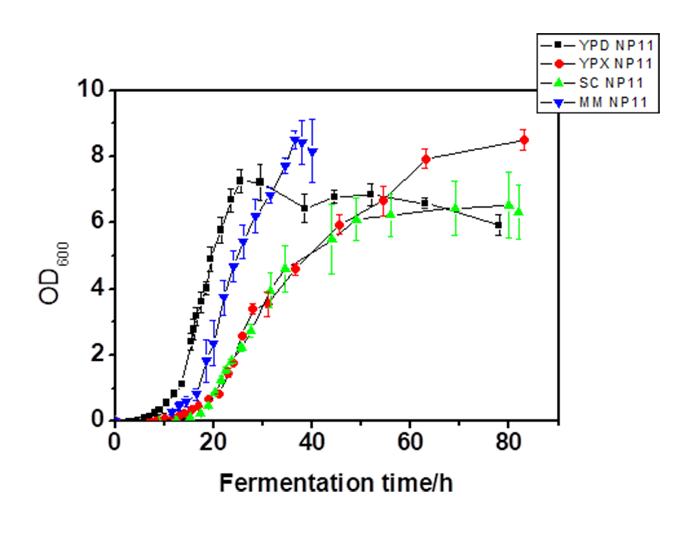


Fig. S1. Cell growth analysis of *R. toruloides* NP11 cultured in different media. The red dot represents YPX medium; black square is YPD medium; blue triangle stands for MM medium and green triangle stands for SC medium. The OD_600_ values of these samples were then measured by a microplate reader. RNA-seq samples were taken at 23 h for YPD, 26 h for YPX, 26 h for SC and 20 h for MM for transcriptome analysis during logarithmic growth phase, and at 48 h for YPD, 60 h for YPX, 48 h for SC and 48 h for MM for transcriptome analysis during stationary growth phase.


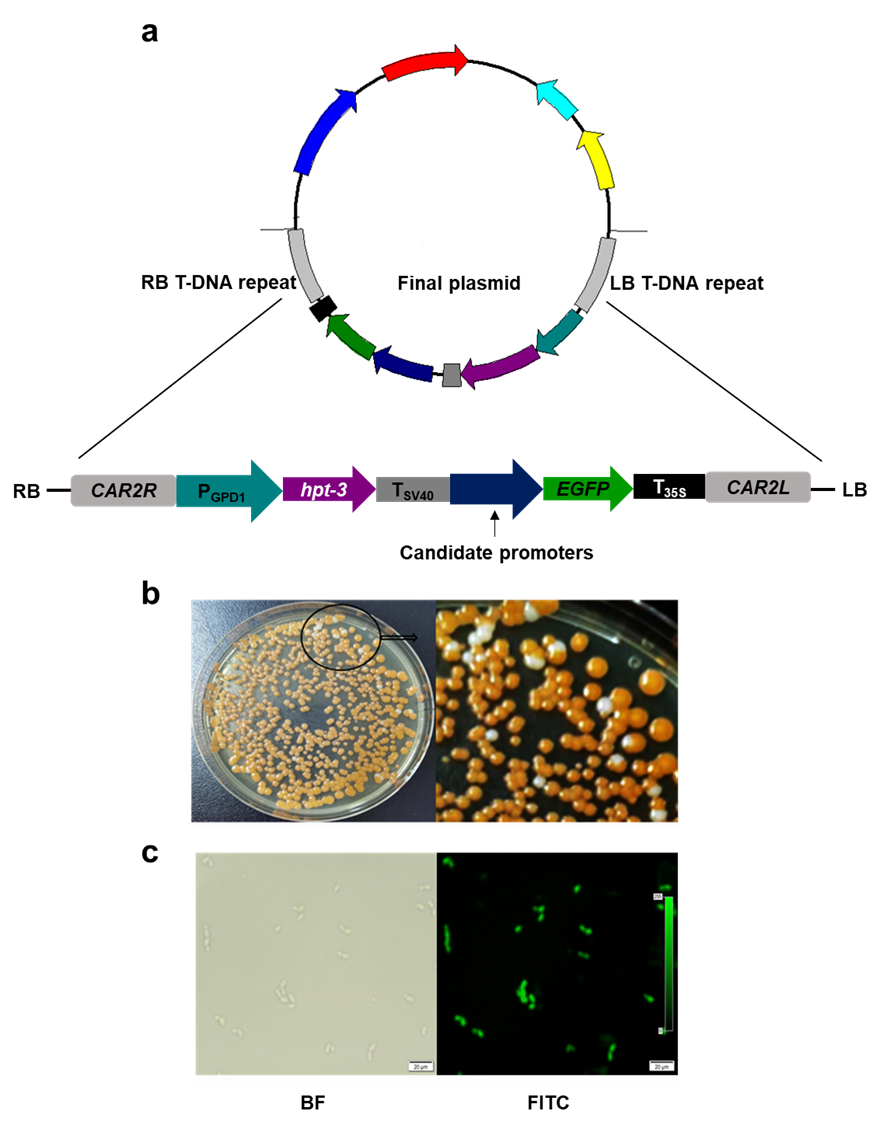


Fig. S2. Plasmid construction and fluorescence images of engineered *R. toruloides*. a) Schematic diagram of plasmids containing candidate promoter-driven *EGFP* cassette. RB/LB, 5’ end and 3’ end of *Agrobacterium tumefaciens T-*DNA; *CAR2R/CAR2L*, right and left homology arms of *CAR2* locus of *R. toruloides*; *P_RgGPD1_*, the glyceraldehyde 3-phosphate promoter of *Rhodotorula graminis* WP1; *P_GPD1_*, the glyceraldehyde 3-phosphate promoter of *R. toruloides* ATCC 10657; *T_SV40_*, transcriptional terminator of *Simian virus* 40; *T_35S_*, transcriptional terminator of 35S; *hpt-3*, codon-optimized hygromycin phosphotransferase gene. b) Image *of R. toruloides* in YPD plate with hygromycin and cefotaxime antibiotics after ATMT transformation. White clones indicated target genes have been successfully inserted to the correct position-*CAR2* locus. c) Fluorescence microscopy images of *R. toruloides* cells. BF, bright filed; FITC, fluorescein isothiocyanate.


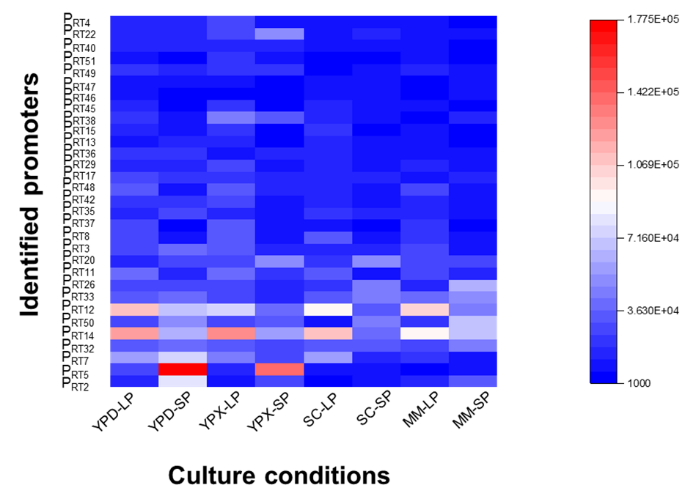


Fig. S3. Expression level of *EGFP* controlled by identified 31 promoters. Fluorescence intensity of EGFP for each promoter was measured by a microplate reader under four different media and two growth phases. The color scale of heatmap is shown in the right bar. Each column represents one culture condition, which is shown below the heatmap. Each row represents a promoter, which is named on the left side of the heatmap. LP: Logarithmic growth phase; SP: Stationary growth phase.


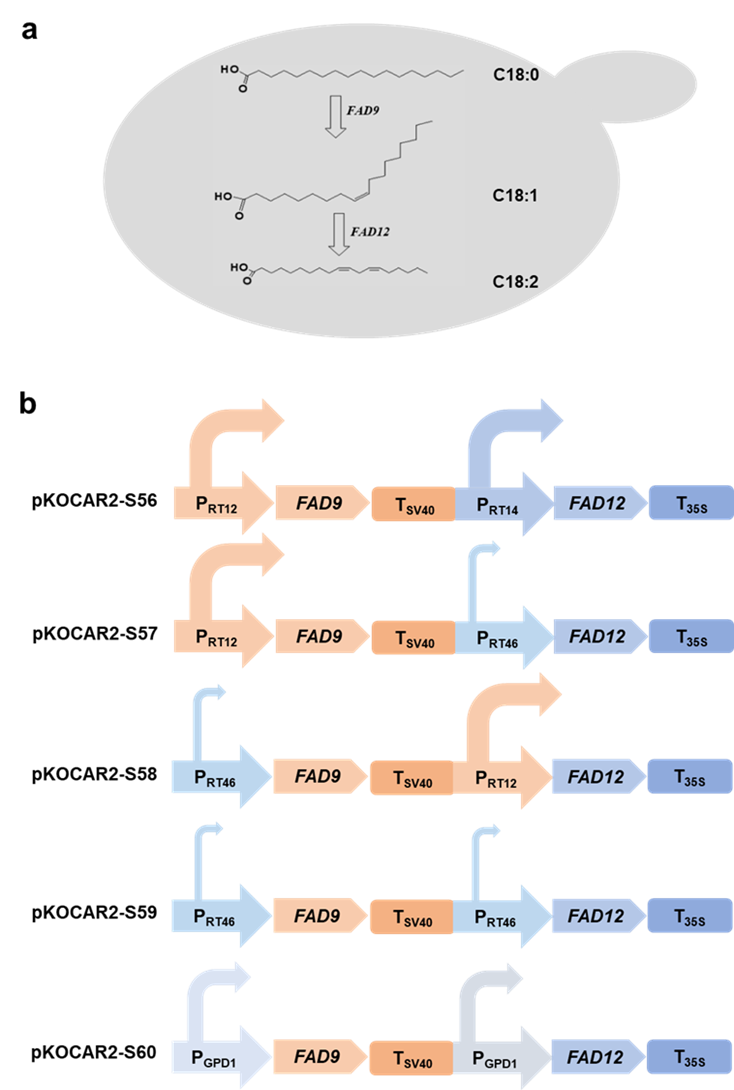


Fig. S4. Combinatorial pathway engineering of linoleic acid production in *R. toruloides*. a) The linoleic acid biosynthetic pathway in *R. toruloides*. FAD9: delta-9 fatty acid desaturase; FAD12: delta-12 fatty acid desaturase. b) Plasmids (from pKOCAR2-S56 to pKOCAR2-S60) with different strength promoters driving *FAD9* and *FAD12* genes. Among them, P_RT12_ and P_RT14_ are extremely strong promoters, and P_RT46_ is a strong promoter. The thickness of the arrow on the promoter corresponds to the strength of the promoter.

Table S1. Strains and plasmids used in this study

| **Strains and plasmids** | **Relevant description** | **Reference** |
| --- | --- | --- |
| **Strains** | | |
| *E. coli* DH5α | *supE*44 *lacU*169 (ϕ80*lacZ*ΔM15) *hsdR*17 *recA*1 *endA*1 *gyrA*96 *thi*-1 *relA*1 |  |
| *R. toruloides* NCYC 1585 | *MAT-A2 leu2- ino* | (Johns et al., 2016) |
| *R. toruloides* NP11 | *MAT A1*, haploid strain | (Wang, Zhang, et al., 2016) |
| *A. tumefaciens* AGL1 | *AGL0 recA::bla pTiBo542ΔT Mop^+^ CbR* | (Wang, Zhang, et al., 2016) |
| *R. toruloides* strain 1 | NCYC 1585 with plasmid pKOCAR2-S1 | This work |
| *R. toruloides* strain 2 | NCYC 1585 with plasmid pKOCAR2-S2 | This work |
| *R. toruloides* strain 3 | NCYC 1585 with plasmid pKOCAR2-S3 | This work |
| *R. toruloides* strain 4 | NCYC 1585 with plasmid pKOCAR2-S4 | This work |
| *R. toruloides* strain 5 | NCYC 1585 with plasmid pKOCAR2-S5 | This work |
| *R. toruloides* strain 6 | NCYC 1585 with plasmid pKOCAR2-S6 | This work |
| *R. toruloides* strain 7 | NCYC 1585 with plasmid pKOCAR2-S7 | This work |
| *R. toruloides* strain 8 | NCYC 1585 with plasmid pKOCAR2-S8 | This work |
| *R. toruloides* strain 9 | NCYC 1585 with plasmid pKOCAR2-S9 | This work |
| *R. toruloides* strain 10 | NCYC 1585 with plasmid pKOCAR2-S10 | This work |
| *R. toruloides* strain 11 | NCYC 1585 with plasmid pKOCAR2-S11 | This work |
| *R. toruloides* strain 12 | NCYC 1585 with plasmid pKOCAR2-S12 | This work |
| *R. toruloides* strain 13 | NCYC 1585 with plasmid pKOCAR2-S13 | This work |
| *R. toruloides* strain 14 | NCYC 1585 with plasmid pKOCAR2-S14 | This work |
| *R. toruloides* strain 15 | NCYC 1585 with plasmid pKOCAR2-S15 | This work |
| *R. toruloides* strain 16 | NCYC 1585 with plasmid pKOCAR2-S16 | This work |
| *R. toruloides* strain 17 | NCYC 1585 with plasmid pKOCAR2-S17 | This work |
| *R. toruloides* strain 18 | NCYC 1585 with plasmid pKOCAR2-S18 | This work |
| *R. toruloides* strain 19 | NCYC 1585 with plasmid pKOCAR2-S19 | This work |
| *R. toruloides* strain 20 | NCYC 1585 with plasmid pKOCAR2-S20 | This work |
| *R. toruloides* strain 22 | NCYC 1585 with plasmid pKOCAR2-S22 | This work |
| *R. toruloides* strain 23 | NCYC 1585 with plasmid pKOCAR2-S23 | This work |
| *R. toruloides* strain 24 | NCYC 1585 with plasmid pKOCAR2-S24 | This work |
| *R. toruloides* strain 26 | NCYC 1585 with plasmid pKOCAR2-S26 | This work |
| *R. toruloides* strain 27 | NCYC 1585 with plasmid pKOCAR2-S27 | This work |
| *R. toruloides* strain 28 | NCYC 1585 with plasmid pKOCAR2-S28 | This work |
| *R. toruloides* strain 29 | NCYC 1585 with plasmid pKOCAR2-S29 | This work |
| *R. toruloides* strain 31 | NCYC 1585 with plasmid pKOCAR2-S31 | This work |
| *R. toruloides* strain 32 | NCYC 1585 with plasmid pKOCAR2-S32 | This work |
| *R. toruloides* strain 33 | NCYC 1585 with plasmid pKOCAR2-S33 | This work |
| *R. toruloides* strain 34 | NCYC 1585 with plasmid pKOCAR2-S34 | This work |
| *R. toruloides* strain 35 | NCYC 1585 with plasmid pKOCAR2-S35 | This work |
| *R. toruloides* strain 36 | NCYC 1585 with plasmid pKOCAR2-S36 | This work |
| *R. toruloides* strain 37 | NCYC 1585 with plasmid pKOCAR2-S37 | This work |
| *R. toruloides* strain 38 | NCYC 1585 with plasmid pKOCAR2-S38 | This work |
| *R. toruloides* strain 39 | NCYC 1585 with plasmid pKOCAR2-S39 | This work |
| *R. toruloides* strain 40 | NCYC 1585 with plasmid pKOCAR2-S40 | This work |
| *R. toruloides* strain 41 | NCYC 1585 with plasmid pKOCAR2-S41 | This work |
| *R. toruloides* strain 42 | NCYC 1585 with plasmid pKOCAR2-S42 | This work |
| *R. toruloides* strain 43 | NCYC 1585 with plasmid pKOCAR2-S43 | This work |
| *R. toruloides* strain 44 | NCYC 1585 with plasmid pKOCAR2-S44 | This work |
| *R. toruloides* strain 45 | NCYC 1585 with plasmid pKOCAR2-S45 | This work |
| *R. toruloides* strain 46 | NCYC 1585 with plasmid pKOCAR2-S46 | This work |
| *R. toruloides* strain 47 | NCYC 1585 with plasmid pKOCAR2-S47 | This work |
| *R. toruloides* strain 48 | NCYC 1585 with plasmid pKOCAR2-S48 | This work |
| *R. toruloides* strain 49 | NCYC 1585 with plasmid pKOCAR2-S49 | This work |
| *R. toruloides* strain 50 | NCYC 1585 with plasmid pKOCAR2-S50 | This work |
| *R. toruloides* strain 51 | NCYC 1585 with plasmid pKOCAR2-S51 | This work |
| *R. toruloides* strain 52 | NCYC 1585 with plasmid pKOCAR2-S52 | This work |
| *R. toruloides* strain 53 | NCYC 1585 with plasmid pKOCAR2-P_GPD1_ | This work |
| *R. toruloides* strain 54 | NCYC 1585 with plasmid pKOCAR2-P_FAS1_ | This work |
| *R. toruloides* strain 55 | NCYC 1585 with plasmid pKOCAR2-P_TPI_ | This work |
| *R. toruloides* strain 56 | NCYC 1585 with plasmid pKOCAR2-S56 | This work |
| *R. toruloides* strain 57 | NCYC 1585 with plasmid pKOCAR2-S57 | This work |
| *R. toruloides* strain 58 | NCYC 1585 with plasmid pKOCAR2-S58 | This work |
| *R. toruloides* strain 59 | NCYC 1585 with plasmid pKOCAR2-S59 | This work |
| *R. toruloides* strain 60 | NCYC 1585 with plasmid pKOCAR2-S60 | This work |
| **Plasmids** | | |
| pKOCAR2 | CAR2L-P_Rg3GPD1_-*hpt-3*-T_sv40_-P_GPD1_-T_35S_-CAR2R | (Koh et al., 2014) |
| pKOCAR2-P_GPD1_ | CAR2L-P_Rg3GPD1_-*hpt-3*-T_sv40_-P_GPD1_-*EGFP*-T_35S_-CAR2R | This work |
| pKOCAR2-S1 | CAR2L-P_Rg3GPD1_-*hpt-3*-T_sv40_-P_RT1_-*EGFP*-T_35S_-CAR2R | This work |
| pKOCAR2-S2 | CAR2L-P_Rg3GPD1_-*hpt-3*-T_sv40_-P_RT2_-*EGFP*-T_35S_-CAR2R | This work |
| pKOCAR2-S3 | CAR2L-P_Rg3GPD1_-*hpt-3*-T_sv40_-P_RT3_-*EGFP*-T_35S_-CAR2R | This work |
| pKOCAR2-S4 | CAR2L-P_Rg3GPD1_-*hpt-3*-T_sv40_-P_RT4_-*EGFP*-T_35S_-CAR2R | This work |
| pKOCAR2-S5 | CAR2L-P_Rg3GPD1_-*hpt-3*-T_sv40_-P_RT5_-*EGFP*-T_35S_-CAR2R | This work |
| pKOCAR2-S6 | CAR2L-P_Rg3GPD1_-*hpt-3*-T_sv40_-P_RT6_-*EGFP*-T_35S_-CAR2R | This work |
| pKOCAR2-S7 | CAR2L-P_Rg3GPD1_-*hpt-3*-T_sv40_-P_RT7_-*EGFP*-T_35S_-CAR2R | This work |
| pKOCAR2-S8 | CAR2L-P_Rg3GPD1_-*hpt-3*-T_sv40_-P_RT8_-*EGFP*-T_35S_-CAR2R | This work |
| pKOCAR2-S9 | CAR2L-P_Rg3GPD1_-*hpt-3*-T_sv40_-P_RT9_-*EGFP*-T_35S_-CAR2R | This work |
| pKOCAR2-S10 | CAR2L-P_Rg3GPD1_-*hpt-3*-T_sv40_-P_RT10_-*EGFP*-T_35S_-CAR2R | This work |
| pKOCAR2-S11 | CAR2L-P_Rg3GPD1_-*hpt-3*-T_sv40_-P_RT11_-*EGFP*-T_35S_-CAR2R | This work |
| pKOCAR2-S12 | CAR2L-P_Rg3GPD1_-*hpt-3*-T_sv40_-P_RT12_-*EGFP*-T_35S_-CAR2R | This work |
| pKOCAR2-S13 | CAR2L-P_Rg3GPD1_-*hpt-3*-T_sv40_-P_RT13_-*EGFP*-T_35S_-CAR2R | This work |
| pKOCAR2-S14 | CAR2L-P_Rg3GPD1_-*hpt-3*-T_sv40_-P_RT14_-*EGFP*-T_35S_-CAR2R | This work |
| pKOCAR2-S15 | CAR2L-P_Rg3GPD1_-*hpt-3*-T_sv40_-P_RT15_-*EGFP*-T_35S_-CAR2R | This work |
| pKOCAR2-S16 | CAR2L-P_Rg3GPD1_-*hpt-3*-T_sv40_-P_RT16_-*EGFP*-T_35S_-CAR2R | This work |
| pKOCAR2-S17 | CAR2L-P_Rg3GPD1_-*hpt-3*-T_sv40_-P_RT17_-*EGFP*-T_35S_-CAR2R | This work |
| pKOCAR2-S18 | CAR2L-P_Rg3GPD1_-*hpt-3*-T_sv40_-P_RT18_-*EGFP*-T_35S_-CAR2R | This work |
| pKOCAR2-S19 | CAR2L-P_Rg3GPD1_-*hpt-3*-T_sv40_-P_RT19_-*EGFP*-T_35S_-CAR2R | This work |
| pKOCAR2-S20 | CAR2L-P_Rg3GPD1_-*hpt-3*-T_sv40_-P_RT20_-*EGFP*-T_35S_-CAR2R | This work |
| pKOCAR2-S22 | CAR2L-P_Rg3GPD1_-*hpt-3*-T_sv40_-P_RT22_-*EGFP*-T_35S_-CAR2R | This work |
| pKOCAR2-S23 | CAR2L-P_Rg3GPD1_-*hpt-3*-T_sv40_-P_RT23_-*EGFP*-T_35S_-CAR2R | This work |
| pKOCAR2-S24 | CAR2L-P_Rg3GPD1_-*hpt-3*-T_sv40_-P_RT24_-*EGFP*-T_35S_-CAR2R | This work |
| pKOCAR2-S26 | CAR2L-P_Rg3GPD1_-*hpt-3*-T_sv40_-P_RT26_-*EGFP*-T_35S_-CAR2R | This work |
| pKOCAR2-S27 | CAR2L-P_Rg3GPD1_-*hpt-3*-T_sv40_-P_RT27_-*EGFP*-T_35S_-CAR2R | This work |
| pKOCAR2-S28 | CAR2L-P_Rg3GPD1_-*hpt-3*-T_sv40_-P_RT28_-*EGFP*-T_35S_-CAR2R | This work |
| pKOCAR2-S29 | CAR2L-P_Rg3GPD1_-*hpt-3*-T_sv40_-P_RT29_-*EGFP*-T_35S_-CAR2R | This work |
| pKOCAR2-S31 | CAR2L-P_Rg3GPD1_-*hpt-3*-T_sv40_-P_RT31_-*EGFP*-T_35S_-CAR2R | This work |
| pKOCAR2-S32 | CAR2L-P_Rg3GPD1_-*hpt-3*-T_sv40_-P_RT32_-*EGFP*-T_35S_-CAR2R | This work |
| pKOCAR2-S33 | CAR2L-P_Rg3GPD1_-*hpt-3*-T_sv40_-P_RT33_-*EGFP*-T_35S_-CAR2R | This work |
| pKOCAR2-S34 | CAR2L-P_Rg3GPD1_-*hpt-3*-T_sv40_-P_RT34_-*EGFP*-T_35S_-CAR2R | This work |
| pKOCAR2-S35 | CAR2L-P_Rg3GPD1_-*hpt-3*-T_sv40_-P_RT35_-*EGFP*-T_35S_-CAR2R | This work |
| pKOCAR2-S36 | CAR2L-P_Rg3GPD1_-*hpt-3*-T_sv40_-P_RT36_-*EGFP*-T_35S_-CAR2R | This work |
| pKOCAR2-S37 | CAR2L-P_Rg3GPD1_-*hpt-3*-T_sv40_-P_RT37_-*EGFP*-T_35S_-CAR2R | This work |
| pKOCAR2-S38 | CAR2L-P_Rg3GPD1_-*hpt-3*-T_sv40_-P_RT38_-*EGFP*-T_35S_-CAR2R | This work |
| pKOCAR2-S39 | CAR2L-P_Rg3GPD1_-*hpt-3*-T_sv40_-P_RT39_-*EGFP*-T_35S_-CAR2R | This work |
| pKOCAR2-S40 | CAR2L-P_Rg3GPD1_-*hpt-3*-T_sv40_-P_RT40_-*EGFP*-T_35S_-CAR2R | This work |
| pKOCAR2-S41 | CAR2L-P_Rg3GPD1_-*hpt-3*-T_sv40_-P_RT41_-*EGFP*-T_35S_-CAR2R | This work |
| pKOCAR2-S42 | CAR2L-P_Rg3GPD1_-*hpt-3*-T_sv40_-P_RT42_-*EGFP*-T_35S_-CAR2R | This work |
| pKOCAR2-S43 | CAR2L-P_Rg3GPD1_-*hpt-3*-T_sv40_-P_RT43_-*EGFP*-T_35S_-CAR2R | This work |
| pKOCAR2-S44 | CAR2L-P_Rg3GPD1_-*hpt-3*-T_sv40_-P_RT44_-*EGFP*-T_35S_-CAR2R | This work |
| pKOCAR2-S45 | CAR2L-P_Rg3GPD1_-*hpt-3*-T_sv40_-P_RT45_-*EGFP*-T_35S_-CAR2R | This work |
| pKOCAR2-S46 | CAR2L-P_Rg3GPD1_-*hpt-3*-T_sv40_-P_RT46_-*EGFP*-T_35S_-CAR2R | This work |
| pKOCAR2-S47 | CAR2L-P_Rg3GPD1_-*hpt-3*-T_sv40_-P_RT47_-*EGFP*-T_35S_-CAR2R | This work |
| pKOCAR2-S48 | CAR2L-P_Rg3GPD1_-*hpt-3*-T_sv40_-P_RT48_-*EGFP*-T_35S_-CAR2R | This work |
| pKOCAR2-S49 | CAR2L-P_Rg3GPD1_-*hpt-3*-T_sv40_-P_RT49_-*EGFP*-T_35S_-CAR2R | This work |
| pKOCAR2-S50 | CAR2L-P_Rg3GPD1_-*hpt-3*-T_sv40_-P_RT50_-*EGFP*-T_35S_-CAR2R | This work |
| pKOCAR2-S51 | CAR2L-P_Rg3GPD1_-*hpt-3*-T_sv40_-P_RT51_-*EGFP*-T_35S_-CAR2R | This work |
| pKOCAR2-S52 | CAR2L-P_Rg3GPD1_-*hpt-3*-T_sv40_-P_RT52_-*EGFP*-T_35S_-CAR2R | This work |
| pKOCAR2-P_FAS1_ | CAR2L-P_Rg3GPD1_-*hpt-3*-T_sv40_-P_FAS1_-*EGFP*-T_35S_-CAR2R | This work |
| pKOCAR2-P_TPI_ | CAR2L-P_Rg3GPD1_-*hpt-3*-T_sv40_-P_TPI_-*EGFP*-T_35S_-CAR2R | This work |
| pKOCAR2-S56 | P_RT12_-*FAD9*-T_SV40_- P_RT14_-*FAD12*-T_35S_ | This work |
| pKOCAR2-S57 | P_RT12_-*FAD9*-T_SV40_-P_RT46_-*FAD12*-T_35S_ | This work |
| pKOCAR2-S58  pKOCAR2-S59  pKOCAR2-S60 | P_RT46_-*FAD9*-T_SV40_-P_RT12_-*FAD12*-T_35S_  P _RT46_-*FAD9*-T_SV40_-P _RT46_-*FAD12*-T_35S_  P_GPD1_-*FAD9*-T_SV40_-P_GPD1_-*FAD12*-T_35S_ | This work  This work  This work |

Table S2. All primers used in this study

| **Primer name** | **Sequence (5’**- **3’)** | **Target PCR product** |  |  |
| --- | --- | --- | --- | --- |
| 1F | cgtttcccgccttcagttttttactagTTGAACCCGAATGGTGCGCA | P_RT1_ |  |  |
| 1R | TCGAGACCGGATCCGCCATGCGTGTGTGGCTTTGTACGAG |  |  |  |
| 2F | cgtttcccgccttcagttttttactagCCGCTTCCGCTTCGCTCGAA | P_RT2_ |  |  |
| 2R | TCGAGACCGGATCCGCCATGTGTGAACGGTGGGAGGACAA |  |  |  |
| 3F | cgtttcccgccttcagttttttactagTCGAGCGCAATGTCCAGCCT | P_RT3_ |  |  |
| 3R | TCGAGACCGGATCCGCCATGGGCTGAACAAAGTTTTCCTG |  |  |  |
| 4F | cgtttcccgccttcagttttttactagCCGAGACCGTTCGTCCCCAC | P_RT4_ |  |  |
| 4R | TCGAGACCGGATCCGCCATGGGTTGCGTGTGAGGGGGAGC |  |  |  |
| 5F | cgtttcccgccttcagttttttactagCCGCATTGGCGCTCCTCCTG | P_RT5_ |  |  |
| 5R | TCGAGACCGGATCCGCCATGGTCTGTCGAGGAGGAGAGTC |  |  |  |
| 6F | cgtttcccgccttcagttttttactagTCGCAAGTTGCCTTCAGATC | P_RT6_ |  |  |
| 6R | TCGAGACCGGATCCGCCATGGGCTGGCGCTGGTTGGTTGG |  |  |  |
| 7F | cgtttcccgccttcagttttttactagCCTCCCTCCGAGCTGATCTG | P_RT7_ |  |  |
| 7R | TCGAGACCGGATCCGCCATGTGCTTTTGATTGTGCGAGTG |  |  |  |
| 8F | cgtttcccgccttcagttttttactagGAGAGAGGGTGTCGAAGTCG | P_RT8_ |  |  |
| 8R | TCGAGACCGGATCCGCCATGTCTGTGAGGGGAACAGAAAT |  |  |  |
| 9F | cgtttcccgccttcagttttttactagGGAGAAGGAGGCTGGAGACT | P_RT9_ |  |  |
| 9R | TCGAGACCGGATCCGCCATGTGCGTCTGAGCGAAGGCGCT |  |  |  |
| 10F | cgtttcccgccttcagttttttactagAGTCGAAAGTCAGCGGCAGA | P_RT10_ |  |  |
| 10R | TCGAGACCGGATCCGCCATGGGTGATGGGCAGCGCGGGAC |  |  |  |
| 11F | cgtttcccgccttcagttttttactagATCCGTCGCGGGCGGGTCCG | P_RT11_ |  |  |
| 11R | TCGAGACCGGATCCGCCATGCTGTTTAGAACGGGAGGAAC |  |  |  |
| 12F | cgtttcccgccttcagttttttactagTCACCTTCCCGCGAAGGACC | P_RT12_ |  |  |
| 12R | TCGAGACCGGATCCGCCATGTGCTGTAGTCTGGCTTTGAG |  |  |  |
| 13F | cgtttcccgccttcagttttttactagGTAATCTTGTGTCGCTTGCT | P_RT13_ |  |  |
| 13R | TCGAGACCGGATCCGCCATGGGCTGATCTACTGTGCTCTG |  |  |  |
| 14F | cgtttcccgccttcagttttttactagCAATCGTGTGAAGAGCGAAG | P_RT14_ |  |  |
| 14R | TCGAGACCGGATCCGCCATGCTAAGAGGAGGAAAGAAGGA |  |  |  |
| 15F | cgtttcccgccttcagttttttactagGAGCATGGGGGACAATGCAT | P_RT15_ |  |  |
| 15R | TCGAGACCGGATCCGCCATGTGTTGAGTGACGGGGAGGGG |  |  |  |
| 16F | cgtttcccgccttcagttttttactagGGGTATCGAGCGCGCCGTCG | P_RT16_ |  |  |
| 16R | TCGAGACCGGATCCGCCATGGCACTCGGGCTGTTGACTGC |  |  |  |
| 17F | cgtttcccgccttcagttttttactagGGTGAGGGAGGAGAAGGAAG | P_RT17_ |  |  |
| 17R | TCGAGACCGGATCCGCCATGGCTGCGAGGTGAGAATGGGC |  |  |  |
| 18F | cgtttcccgccttcagttttttactagtttccggtcctGCGGACTTC | P_RT18_ |  |  |
| 18R | TCGAGACCGGATCCGCCATGGGTCGCGGGTCGTTGTGCGG |  |  |  |
| 19F | cgtttcccgccttcagttttttactagGAGAGAAGTTGGCGCGAGCA | P_RT19_ |  |  |
| 19R | TCGAGACCGGATCCGCCATGCGAGAGTTGGCGAGTCAGGA |  |  |  |
| 20F | cgtttcccgccttcagttttttactagCCTGGCGCACTGCGAGTCGT | P_RT20_ |  |  |
| 20R | TCGAGACCGGATCCGCCATGCGTGCTGTAGGGGGCCGAGG |  |  |  |
| 21F | cgtttcccgccttcagttttttactagTTCACTACCCTCCCACCTCC | P_RT21_ |  |  |
| 21R | TCGAGACCGGATCCGCCATGGGCTGCGTGAGTGCGCGAGG |  |  |  |
| 22F | cgtttcccgccttcagttttttactagCAAGCGTTCAAGAACTTCCA | P_RT22_ |  |  |
| 22R | TCGAGACCGGATCCGCCATGGGCTGTAACTTAGTTGTTTG |  |  |  |
| 23F | cgtttcccgccttcagttttttactagGCAGCAACAGATGATGTACC | P_RT23_ |  |  |
| 23R | TCGAGACCGGATCCGCCATGGGCGGCTAGCGGGAAGCCAG |  |  |  |
| 24F | cgtttcccgccttcagttttttactagGCCCCAGATCCAATCACTTT | P_RT24_ |  |  |
| 24R | TCGAGACCGGATCCGCCATGCTTGGGCACAAGCAGGCGAA |  |  |  |
| 25F | cgtttcccgccttcagttttttactagCGTCGCGGGACGAGCAGCTC | P_RT25_ |  |  |
| 25R | TCGAGACCGGATCCGCCATGGTGTGTTGGTGGGAAGGACG |  |  |  |
| 26F | cgtttcccgccttcagttttttactagCGCGGCGCGCCATTCGGAAG | P_RT26_ |  |  |
| 26R | TCGAGACCGGATCCGCCATGCTGTGCTGCTCTACTGGCTG |  |  |  |
| 27F | cgtttcccgccttcagttttttactagGCTGCGGCGCAAGTTGCGCG | P_RT27_ |  |  |
| 27R | TCGAGACCGGATCCGCCATGCGTCGCGGAATTCGAGAGGA |  |  |  |
| 28F | cgtttcccgccttcagttttttactagGATGCGCTTGCGGTTCATCG | P_RT28_ |  |  |
| 28R | TCGAGACCGGATCCGCCATGTGAgagtgtgtgagtgacaa |  |  |  |
| 29F | cgtttcccgccttcagttttttactagCGACAGAACGCCGACCATTG | P_RT29_ |  |  |
| 29R | TCGAGACCGGATCCGCCATGTGTCCTTTGCAGTGGTTGGG |  |  |  |
| 30F | cgtttcccgccttcagttttttactagGAACCCGCCCACGCCCACAC | P_RT30_ |  |  |
| 30R | TCGAGACCGGATCCGCCATGTACTGTTCGGTGAGTTGTGG |  |  |  |
| 31F | cgtttcccgccttcagttttttactagCCGTCCTCGAGTACGACTGG | P_RT31_ |  |  |
| 31R | TCGAGACCGGATCCGCCATGGACGGCGAGGCCGATGAGAC |  |  |  |
| 32F | cgtttcccgccttcagttttttactagGATCTGGGAGTGCAAGCCCG | P_RT32_ |  |  |
| 32R | TCGAGACCGGATCCGCCATGCTGCGAGCATCCCAGCGGTT |  |  |  |
| 33F | cgtttcccgccttcagttttttactagTGAACTGTCCGTTGACCAGG | P_RT33_ |  |  |
| 33R | TCGAGACCGGATCCGCCATGGCTGTGAGATGGGCACCGCG |  |  |  |
| 34F | cgtttcccgccttcagttttttactagCGTGGAAAGAGCGGACGAGC | P_RT34_ |  |  |
| 34R | TCGAGACCGGATCCGCCATGCCTTACTGGCGGTGTGGCCT |  |  |  |
| 35F | cgtttcccgccttcagttttttactagCTTGATGACTCGGACAGCGA | P_RT35_ |  |  |
| 35R | TCGAGACCGGATCCGCCATGGGCGGCGATTAAGGACGGTG |  |  |  |
| 36F | cgtttcccgccttcagttttttactagCAACAAGAAGAAGAGGATCA | P_RT36_ |  |  |
| 36R | TCGAGACCGGATCCGCCATGGAAGTGTGCTGGATCAGTAC |  |  |  |
| 37F | cgtttcccgccttcagttttttactagGTGAGGGTCGAACACTCCTT | P_RT37_ |  |  |
| 37R | TCGAGACCGGATCCGCCATGGCTGACTGGGTGATGGCGCG |  |  |  |
| 38F | cgtttcccgccttcagttttttactagAGGTCAATTGCTGAGCAGGC | P_RT38_ |  |  |
| 38R | TCGAGACCGGATCCGCCATGGAGGGGCGGGTTTGTTTGGT |  |  |  |
| 39F | cgtttcccgccttcagttttttactagGCAGAGGGTTTCGACGCTCT | P_RT39_ |  |  |
| 39R | TCGAGACCGGATCCGCCATGGAGGTTGCACGGTCAGTCAT |  |  |  |
| 40F | cgtttcccgccttcagttttttactagGCCTCGATAAAGAACGGGGT | P_RT40_ |  |  |
| 40R | TCGAGACCGGATCCGCCATGTGTGACTGTCGGAGACGTGG |  |  |  |
| 41F | cgtttcccgccttcagttttttactagTGCTCAAGAAGATCCCGGCG | P_RT41_ |  |  |
| 41R | TCGAGACCGGATCCGCCATGCTACTCTGCCGTCACTGATC |  |  |  |
| 42F | cgtttcccgccttcagttttttactagTCGAGCGGACGATCCATACT | P_RT42_ |  |  |
| 42R | TCGAGACCGGATCCGCCATGCGTAAAGGTCGAGAGGGACG |  |  |  |
| 43F | cgtttcccgccttcagttttttactagGCAGAGTACTGACAGAGCTC | P_RT43_ |  |  |
| 43R | TCGAGACCGGATCCGCCATGTTTAAGCTGAACGTTCGAGG |  |  |  |
| 44F | cgtttcccgccttcagttttttactagGCAGCCATCGTTCAGCAGGA | P_RT44_ |  |  |
| 44R | TCGAGACCGGATCCGCCATGGGCGTTTGTTCTGTCGTCTC |  |  |  |
| 45F | cgtttcccgccttcagttttttactagGCGAGCGATACGAGCAAAGA | P_RT45_ |  |  |
| 45R | TCGAGACCGGATCCGCCATGGGCCGAGTGTTGCGCAGAGT |  |  |  |
| 46F | cgtttcccgccttcagttttttactagTCGCCTGGACCAGCGCCGCT | P_RT46_ |  |  |
| 46R | TCGAGACCGGATCCGCCATGCGTAGGAAGTGTAAACGAGT |  |  |  |
| 47F | cgtttcccgccttcagttttttactagGAGCGGCAGGCCAAGCGGCG | P_RT47_ |  |  |
| 47R | TCGAGACCGGATCCGCCATGTGTAGTGTGTGGTAGCAAGT |  |  |  |
| 48F | cgtttcccgccttcagttttttactagCGAGACGTCGCGACGAGCGG | P_RT48_ |  |  |
| 48R | TCGAGACCGGATCCGCCATGCTTCTGGTGGAGCACGCGGG |  |  |  |
| 49F | cgtttcccgccttcagttttttactagGATGGAGCTTTTGCTGGGCG | P_RT49_ |  |  |
| 49R | TCGAGACCGGATCCGCCATGGGTGGCCTGTCGGGGCAGTC |  |  |  |
| 50F | cgtttcccgccttcagttttttactagCTCGACGCCACGGTAGGAGA | P_RT50_ |  |  |
| 50R | TCGAGACCGGATCCGCCATGCTGCGTAGTGGACAAGAGTG |  |  |  |
| 51F | cgtttcccgccttcagttttttactagAGCGCATGCGGCCGGTACCG | P_RT51_ |  |  |
| 51R | TCGAGACCGGATCCGCCATGTGTTAGCTTGGCCTACACAA |  |  |  |
| 52F | cgtttcccgccttcagttttttactagGTGCAAGTCCTCGCGCGACG | P_RT52_ |  |  |
| 52R | TCGAGACCGGATCCGCCATGCTTAGAGCGTAGGCGATGCA |  |  |  |
| FAS1F | cgtttcccgccttcagttttttactagAGGGGAGGTCATCCGCGTTC | P_FAS1_ |  |  |
| FAS1R | TCGAGACCGGATCCGCCATGCCTGTGCGGTGTTTGACGAG |  |  |  |
| TPIF | cgtttcccgccttcagttttttactagCGGAGCGTCCATTCGCTCCC | P_TPI_ |  |  |
| TPIR | TCGAGACCGGATCCGCCATGGGTTGCTTAGGTGCGGATAG |  |  |  |
| 12F1 | ccttcagttttttactagtGTCACCTTCCCGCGAAGGACC | P_RT12_ | |  |
| 12R1 | GTGCCGAAGAGGCAGTCATGATATCTGCTGTAGTCTGGCTTTGAG |  |  |  |
| FAD9F1 | ATGACTGCCTCTTCGGCACT | FAD9 | |  |
| FAD9R1 | TTACGCCTTGACCTTCAGGC |  |  |  |
| SV40F1 | GCCTGAAGGTCAAGGCGTAAatctaagatacattgatgagtttggacaaa | T_SV40_ | |  |
| SV40R1 | CACACGATTGTTGTTTAAAtttaccacatttgtagaggttttacttgct |  |  |  |
| 14F1 | cctctacaaatgtggtaaaTTTAAACAACAATCGTGTG | P_RT14_ | |  |
| 14R1 | GAGGGTGGCGGCCATCCCGGGCTAAGAGGAGGAAAGAAGG |  |  |  |
| FAD12F1 | ATGGCCGCCACCCTCCGCCA | FAD12 | |  |
| FAD12R1 | GCTTGTCGATCGACAGATCCCTAGAGTCCCTCGACGCCCG |  |  |  |
| 12F2 | ccttcagttttttactagtGTCACCTTCCCGCGAAGGACC | P_RT12_ | |  |
| 12R2 | GTGCCGAAGAGGCAGTCATGATATCTGCTGTAGTCTGGCTTTGAG |  |  |  |
| FAD9F2 | ATGACTGCCTCTTCGGCACT | FAD9 | |  |
| FAD9R2 | TTACGCCTTGACCTTCAGGC |  |  |  |
| SV40F2 | GCCTGAAGGTCAAGGCGTAAatctaagatacattgatgagtttggacaaa | T_SV40_ | |  |
| SV40R2 | GGTCCAGGCGATTGTTTAAACtttaccacatttgtagagg |  |  |  |
| 43F2 | ctacaaatgtggtaaaGTTTAAACAATCGCCTGGACCAGCGCCGCT | P_RT46_ | |  |
| 43R2 | GAGGGTGGCGGCCATCCCGGGCGTAGGAAGTGTAAACGAGT |  |  |  |
| FAD12F2 | ATGGCCGCCACCCTCCGCCA | FAD12 | |  |
| FAD12R2 | GCTTGTCGATCGACAGATCCCTAGAGTCCCTCGACGCCCG |  |  |  |
| 43F3 | ccttcagttttttactagtGTCGCCTGGACCAGCGCCGCT | P_RT46_ | |  |
| 43R3 | GAAGAGGCAGTCATGATATCCGTAGGAAGTGTAAACGAGT |  |  |  |
| FAD9F3 | ATGACTGCCTCTTCGGCACT | FAD9 | |  |
| FAD9R3 | TTACGCCTTGACCTTCAGGC |  |  |  |
| SV40F3 | GCCTGAAGGTCAAGGCGTAAatctaagatacattgatgag | T_SV40_ | |  |
| SV40R3 | CACACGATTGTTGTTTAAAtttaccacatttgtagagg |  |  |  |
| 12F3 | cctctacaaatgtggtaaaTTTAAACAACAATCGTGTG | P_RT12_ | |  |
| 12R3 | GAGGGTGGCGGCCATCCCGGGCTAAGAGGAGGAAAGAAGG |  |  |  |
| FAD12F3 | ATGGCCGCCACCCTCCGCCA | FAD12 | |  |
| FAD12R3 | GCTTGTCGATCGACAGATCCCTAGAGTCCCTCGACGCCCG |  |  |  |
| 43F4 | ccttcagttttttactagtGTCGCCTGGACCAGCGCCGCT | P_RT46_ | |  |
| 43R4 | GAAGAGGCAGTCATGATATCCGTAGGAAGTGTAAACGAGT |  |  |  |
| FAD9F4 | ATGACTGCCTCTTCGGCACT | FAD9 | |  |
| FAD9R4 | TTACGCCTTGACCTTCAGGC |  |  |  |
| SV40F4 | GCCTGAAGGTCAAGGCGTAAatctaagatacattgatgag | T_SV40_ | |  |
| SV40R4 | GTCCAGGCGATTGTTTAAACtttaccacatttgtagaggt |  |  |  |
| 43F5 | ctacaaatgtggtaaaGTTTAAACAATCGCCTGGACCAGCGCCGCT | P_RT46_ | |  |
| 43R5 | GAGGGTGGCGGCCATCCCGGGCGTAGGAAGTGTAAACGAGT |  |  |  |
| FAD12F4 | ATGGCCGCCACCCTCCGCCA | FAD12 | |  |
| FAD12R4 | GCTTGTCGATCGACAGATCCCTAGAGTCCCTCGACGCCCG |  |  |  |
| GPD1F1 | gccttcagttttttactagtGGACGGCTTGTTCTCTCCTG | P_GPD1_ | |  |
| GPD1R1 | GAAGAGGCAGTCATGATATCgtgagtgatctggtgttgtt |  |  |  |
| FAD9F5 | accagatcactcacGATATCATGACTGCCTCTTCGGCACT | FAD9 | |  |
| FAD9R5 | ctcatcaatgtatcttagatTTACGCCTTGACCTTCAGGC |  |  |  |
| SV40F5 | GCCTGAAGGTCAAGGCGTAAatctaagatacattgatgag | T_SV40_ | |  |
| SV40R5 | CACACGATTGTTGTTTAAAtttaccacatttgtagagg |  |  |  |
| GPD1F2 | caaatgtggtaaaGTTTAAACAAGACGGCTTGTTCTCTCCTG | P_GPD1_ | |  |
| GPD1R2 | GAGGGTGGCGGCCATCCCGGGgtgagtgatctggtgttgtt |  |  |  |
| FAD12F5 | accagatcactcacCCCGGGATGGCCGCCACCCTCCGCCA | FAD12 | |  |
| FAD12R5 | GCTTGTCGATCGACAGATCCCTAGAGTCCCTCGACGCCCG |  |  |  |

Table S3. Gene ID (description of the ID can be found in NCBI database: https://www.ncbi.nlm.nih.gov/gene/) of the top 15 genes with the highest expression levels under different culture conditions at logarithmic growth phase and stationary growth phase: 23 h and 48 h for YPD (YPD23 and YPD48), 26 h and 60 h for YPX (YPX26 and YPX60), 26 h and 48 h for SC (SC26 and SC48), 20 h and 48 h for MM (MM20 and MM48). A total of 52 candidate genes were obtained after removing the duplicates, marked in bold. By blasting these genes in NCBI (https://www.ncbi.nlm.nih.gov/assembly/GCA_000320785.2), we obtained the scaffold sequences where these genes are located.

| **YPD48** | **YPD23** | **YPX60** | **YPX26** | **SC26** | **SC48** | **MM20** | **MM48** |
| --- | --- | --- | --- | --- | --- | --- | --- |
| **27368476** | 27369119 | 27367113 | **27371047** | 27369119 | 27370552 | 27369119 | **27365369** |
| **27368376** | 27369110 | 27369119 | 27369119 | 27369110 | 27369119 | 27369110 | **27365895** |
| **27369119** | 27371855 | 27364022 | 27369110 | 27368376 | 27368163 | 27368376 | **27369851** |
| **27367219** | 27368476 | 27368376 | 27371855 | 27364137 | **27366763** | 27367219 | **27368163** |
| **27365962** | 27367219 | 27367219 | 27367219 | 27367219 | 27369110 | 27368476 | **27370093** |
| **27369132** | 27368376 | 27371855 | 27368829 | **27365879** | **27368829** | **27366801** | **27368700** |
| **27371855** | **27370552** | 27369110 | **27364761** | **27366699** | 27368376 | 27364137 | **27367075** |
| **27371881** | **27371704** | 27369132 | **27371731** | 27367113 | 27364645 | 27371704 | 27371855 |
| **27367113** | 27369132 | 27367075 | **27368684** | 27371855 | **27366188** | 27371855 | **27372128** |
| **27369661** | 27369661 | 27370537 | **27369947** | 27370419 | **27366128** | 27370552 | **27370537** |
| **27364645** | 27369809 | 27365962 | 27369132 | 27369132 | 27369132 | **27367759** | **27366037** |
| **27369809** | 27364137 | 27371881 | **27366710** | 27370552 | 27369930 | 27369132 | **27371430** |
| **27370419** | 27371881 | **27368196** | **27369450** | 27369809 | 27369809 | 27371881 | 27367113 |
| **27369110** | **27364022** | **27369727** | **27364309** | **27371760** | 27367113 | **27364336** | 27364137 |
| **27364137** | **27369930** | **27371661** | 27369661 | 27371881 | **27371268** | 27369661 | 27368476 |

Table S4. Summary of the cloning and evaluation results of 52 candidate promoters. Among them, 3 promoter sequences were not successfully amplified from *R. toruloides* genome; 11 promoters did not generate any EGFP fluorescence; 7 promoters drove very weak EGFP fluorescence expression and no further study was performed; 31 promoters driving high EGFP expression were further investigated. The ID of the promoter’s corresponding gene can be obtained on NCBI website.

| **Unsuccessfully amplified** | | | **Without fluorescent signal** | | | **With very weak fluorescent signal** | | | **With strong fluorescent signal** | | | | | |  |
| --- | --- | --- | --- | --- | --- | --- | --- | --- | --- | --- | --- | --- | --- | --- | --- |
| **Promoter**  **name** | | **ID of the corresponding gene** | | **Promoter**  **name** | **ID of the corresponding gene** | | **Promoter name** | **ID of the corresponding gene** | **Promoter**  **name** | **ID of the corresponding gene** | **Promoter**  **name** | **ID of the corresponding gene** | **Promoter**  **name** | **ID of the corresponding gene** |  |
| P_RT21_ | | 27364761 | | P_RT6_ | 27369132 | | P_RT1_ | 27368476 | P_RT2_ | 27368376 | P_RT3_ | 27369119 | P_RT13_ | 27370419 |  |
| P_RT25_ | | 27366710 | | P_RT9_ | 27367113 | | P_RT28_ | 27368196 | P_RT7_ | 27371855 | P_RT8_ | 27371881 | P_RT15_ | 27364137 |  |
| P_RT30_ | | 27371661 | | P_RT10_ | 27369661 | | P_RT27_ | 27369727 | P_RT5_ | 27365962 | P_RT37_ | 27368163 | P_RT38_ | 27370093 |  |
|  | |  | | P_RT16_ | 27370552 | | P_RT39_ | 27368700 | P_RT32_ | 27367759 | P_RT35_ | 27365895 | P_RT22_ | 27371731 |  |
|  | |  | | P_RT18_ | 27364022 | | P_RT41_ | 27372128 | P_RT14_ | 27369110 | P_RT42_ | 27370537 | P_RT4_ | 27367219 |  |
|  | |  | | P_RT19_ | 27369930 | | P_RT43_ | 27366037 | P_RT50_ | 27366188 | P_RT48_ | 27366763 | P_RT40_ | 27367075 |  |
|  | |  | | P_RT23_ | 27368684 | | P_RT44_ | 27371430 | P_RT12_ | 27369809 | P_RT17_ | 27371704 | P_RT45_ | 27365879 |  |
|  | |  | | P_RT24_ | 27369947 | |  |  | P_RT33_ | 27364336 | P_RT36_ | 27369851 | P_RT46_ | 27366699 |  |
|  |  | | | P_RT31_ | 27366801 | |  |  | P_RT20_ | 27371047 | P_RT29_ | 27364309 | P_RT47_ | 27371760 |  |
|  | |  | | | P_RT34_ | 27365369 | |  |  | P_RT11_ | 27364645 |  |  | P_RT49_ | 27368829 |
|  |  | | | P_RT52_ | 27371268 | |  |  | P_RT26_ | 27369450 |  |  | P_RT51_ | 27366128 |  |

Table S5. The sequence analysis of promoters failed to drive EGFP expression. The highlighted font are the possible G/GT and AG/G splice sites of introns (Chung et al., 2006) between the 3' end of the promoter (the black font) and the first 22 bp of *EGFP* gene (the green font).

| **Promoters** | **Sequence analysis** |
| --- | --- |
| P_RT6_ | ATGGGTGTGCGTGCCACACACTCTCTCAGACTAGACGCACACTGACGAGCCACCCACGCCACGCAGATGGCGGATCCGGTCTCGAAGG |
| P_RT10_ | ATGAGTTGAATACAAACTCGGGTTCAATACAAAGTGCCGTCTCCAGCCCGTGCCCTCTCGCCCTCCCTCCCCGTCCCGCGCTGCCCATCACCATGGCGGATCCGGTCTCGAAGG |
| P_RT16_ | ATGAGGAGGAGGTGAGAGAAGGCGGGAGTTGAGTGGGACTGACTGTGCGTGAGCTCTGCGTTGTCCATAAGTGTCCCTGCACACACTCGCACTCCCTCGCTGGCTCTCGCAGTCAACAGCCCGAGTGCATGGCGGATCCGGTCTCGAAGG |
| P_RT19_ | ATGGGACGCGGTACGTAGGCGCAGGCGCGAGGGACGGGCGACTTCACGAACTGACCCTTTGCCCCCTTCCTCCCGCATACACCACCTCCTGACTCGCCAACTCTCGATGGCGGATCCGGTCTCGAAGGGCGAGG |
| P_RT23_ | ATGTCAAAGGCTGGTCCTCCTCCTGCAATCGAGTCTTCCTGCATATCCAAGCAACGCTCCCTCAAGCTGCTCGTTACCAGCCCTCGCTTGCCGTCGGCCCCACTGCCCTCGGCACCTCTTGAGCGCTATCGCTCTCCTGGCTTCCCGCTAGCCGCCATGGCGGATCCGGTCTCGAAGG |
| P_RT31_ | ATGGTGCGTTGCGCACGAGTTGCCGACGACTTGTTCCGAAACCGAGAGTTGACGGATCTCGCTTTCCGCAATGGCGGATCCGGTCTCGAAGG |

Table S6. Summary of the relative strengths of known promoters and our identified five new promoters native to *R. toruloides*. The ID of the promoter’s corresponding gene can be accessed via the NCBI database (<https://www.ncbi.nlm.nih.gov/gene/>).

| **Promoter name** | **ID of the corresponding gene** | **Corresponding gene’s function** | **Promoter strength compared to P_GPD1_** | **Conditions** | **Reference** |
| --- | --- | --- | --- | --- | --- |
| P_GPD1_  (also named P7 in the work of Nora et al. 2019) | KB722658.1 | Glyceraldehyde-3-phosphate dehydrogenase | Strong promoter | Driving *RtGFP* and *hpt-3* genes in YPD medium | (Liu et al., 2013) |
| P_LDP1_ | RHTO_05627 | Lipid droplet protein | 4-11 folds | Driving luciferase reporter gene *RtLUC2* in YPD medium | (Liu et al., 2016) |
| P_FAS1_ | --- | Fatty acid synthase β subunit gene | 20-90% | Same as above | (Liu et al., 2016) |
| P_ACC1_ | --- | Acetyl-CoA carboxylase | 50-160% | Same as above | (Liu et al., 2016) |
| P_PGI_ | KB722666.1 | Glucose 6-phosphate isomerase | ~4 folds | Driving *hpt-3* gene in YPD medium | (Wang, Lin, et al., 2016) |
| P_PGK_ | KB722642.1 | Phosphoglycerate kinase | ~2 folds | Same as above | (Wang, Lin, et al., 2016) |
| P_FBA_ | KB722643.1 | Fructose 1,6-biphosphatealdolase | <2 folds | Same as above | (Wang, Lin, et al., 2016) |
| P_TPI_ | KB722654.1 | Triose phosphate isomerase | ~1.2 folds | Same as above | (Wang, Lin, et al., 2016) |
| P_RT14_ | 27369110 | Elongation factor EF-1 alpha subunit | 2.3-8.7 folds | Driving *EGFP* gene with 2.3-fold fluorescence in stationary phase when cultured in MM medium, and 8.7-fold fluorescence in log phase when cultured in YPX medium | This work |
| P_RT32_ | 27367759 | Glyceraldehyde-3-phosphate dehydrogenase | 1.2-3.4 folds | Driving *EGFP* gene with  1.2-fold fluorescence in log phase when cultured in YPD medium and 3.4-fold in stationary phase when cultured in YPX medium | This work |
| P_RT50_ | 27366188 | 60S acidic ribosomal protein p2 | 1.7-6.8 folds | Driving *EGFP* gene with 1.7-fold fluorescence in log phase when cultured in YPX medium, and 6.8-folds fluorescence in stationary phase when cultured in MM medium | This work |
| P_RT5_ | 27365962 | --- | 19 folds | Driving *EGFP* gene in stationary phase when cultured in YPX medium | This work |
| P_RT45_ | 27365879 | Plasma membrane H^+^- transporting ATPase | 10% | Driving *EGFP* gene in stationary phase when cultured in MM medium | This work |

Table S7. List of the overlapped promoters identified in this study with the previous reported work. Compared to the previous work (Nora et al., 2019), seven promoters characterized in this work are overlapped. However, the sequences and strength of these promoters are not the same due to the different definition in the promoter regions. The protein ID refers to the *R. toruloides* IFO0880 v4.0 genome sequence, which can be found on the Joint Genome Institute (JGI) MycoCosm site:[https://genome.jgi.doe.gov/ Rhoto_IFO0880_4/ Rhoto_IFO0880_4. home.html](https://genome.jgi.doe.gov/%20Rhoto_IFO0880_4/%20Rhoto_IFO0880_4.%20home.html).

| **Name of previously reported promoters** | **Protein ID** | **Name of promoters in this work** |
| --- | --- | --- |
| P7 | 10613 | P_RT32_ |
| P14 | 12693 | P_RT14_ |
| P19 | 16418 | P_RT50_ |
| P15 | 12704 | P_RT3_ |
| P8 | 8752 | P_RT51_ |
| P17 | 15825 | P_RT15_ |
| P18 | 11331 | P_RT13_ |

**References**

Chung, B. Y., Simons, C., Firth, A. E., Brown, C. M., Hellens, R. P. (2006). Effect of 5'UTR introns on gene expression in *Arabidopsis thaliana*. *BMC Genomics, 7*, 120. doi:10.1186/1471-2164-7-120

Johns, A. M., Love, J., Aves, S. J. (2016). Four Inducible Promoters for Controlled Gene Expression in the Oleaginous Yeast *Rhodotorula toruloides*. *Front Microbiol, 7*, 1666. doi:10.3389/fmicb.2016.01666

Koh, C. M., Liu, Y., Moehninsi, Du, M., Ji, L. (2014). Molecular characterization of KU70 and KU80 homologues and exploitation of a KU70-deficient mutant for improving gene deletion frequency in *Rhodosporidium toruloides*. *BMC Microbiol, 14*, 50. doi:10.1186/1471-2180-14-50

Liu, Y., Koh, C. M., Sun, L., Hlaing, M. M., Du, M., Peng, N., Ji, L. (2013). Characterization of glyceraldehyde-3-phosphate dehydrogenase gene RtGPD1 and development of genetic transformation method by dominant selection in oleaginous yeast *Rhodosporidium toruloides*. *Appl Microbiol Biotechnol, 97*, 719-729. doi:10.1007/s00253-012-4223-9

Liu, Y., Yap, S. A., Koh, C. M., Ji, L. (2016). Developing a set of strong intronic promoters for robust metabolic engineering in oleaginous *Rhodotorula* (*Rhodosporidium*) yeast species. *Microb Cell Fact, 15*, 200. doi:10.1186/s12934-016-0600-x

Nora, L. C., Wehrs, M., Kim, J., Cheng, J. F., Tarver, A., Simmons, B. A., Magnuson, J., Harmon-Smith, M., Silva-Rocha, R., Gladden, J. M., Mukhopadhyay, A., Skerker, J. M., Kirby, J. (2019). A toolset of constitutive promoters for metabolic engineering of *Rhodosporidium toruloides*. *Microb Cell Fact, 18*, 117. doi:10.1186/s12934-019-1167-0

Wang, Y., Lin, X., Zhang, S., Sun, W., Ma, S., Zhao, Z. K. (2016). Cloning and evaluation of different constitutive promoters in the oleaginous yeast *Rhodosporidium toruloides*. *Yeast, 33*, 99-106. doi:10.1002/yea.3145

Wang, Y., Zhang, S., Potter, M., Sun, W., Li, L., Yang, X., Jiao, X., Zhao, Z. K. (2016). Overexpression of Delta12-Fatty Acid Desaturase in the Oleaginous Yeast *Rhodosporidium toruloides* for Production of Linoleic Acid-Rich Lipids. *Appl Biochem Biotechnol, 180*, 1497-1507. doi:10.1007/s12010-016-2182-9
